# XPRESSO: Rapid genetic engineering of human pluripotent stem cells for durable overexpression using a modular anti-silencing vector

**DOI:** 10.1016/j.stemcr.2025.102603

**Published:** 2025-08-21

**Authors:** Yehuda Wexler, Harel Grinstein, Irit Huber, Shany Glatstein, Matteo Ghiringhelli, Oded Edri, Michal Landesberg, Daniel Shiff, Gil Arbel, Idan Rosh, Ashwani Choudhary, Shani Stern, Lior Gepstein

**Affiliations:** 1 Sohnis Research Laboratory for Cardiac Electrophysiology and Regenerative Medicine, the Rappaport Faculty of Medicine and Research Institute, Technion‒Israel Institute of Technology, POB 9649, Haifa 3109601, Israel; 2 Sagol Department of Neurobiology Faculty of Natural Sciences, University of Haifa, Haifa 3498838, Israel; 3 Cardiology Department, Rambam Health Care Campus, 8 Haliya Hasniya St, Haifa 3109601, Israel

**Keywords:** pluripotent stem cells, genetic engineering, hiPSC-CMs, optogenetics, transgene silencing, CRISPR-Cas9, sleeping beauty transposon, epigenetic silencing, cardiomyocytes, neurons

## Abstract

Ectopic expression of proteins in human pluripotent stem cells (hPSCs) is highly desirable as a research tool and important for clinical translation. However, genetically engineering hPSCs for long-term overexpression of proteins remains inefficient, labor-intensive, and plagued by epigenetic silencing, necessitating dedication of significant resources, and entailing laborious workflows. To address these limitations, we report the development of XPRESSO (expedited persistent and robust engineering of stem cells with sleeping beauty for overexpression), a modular “anti-silencing” transposon vector, which we have combined with a highly efficient and accessible methodology for the rapid generation of genetically modified hPSC lines in a gene-independent manner. Using this method, we successfully generated dozens of stable hPSC lines with robust and continuous functional expression of optogenetic proteins, Cas9, shRNA, and a calcium indicator in both undifferentiated and differentiated (cardiomyocyte and neuronal) cells.

## Highlights

• XPRESSO is a modular SB transposon engineered for stable overexpression in hPSCs
• It is rapid and transgene-independent as shown with an optogenetic channel and Cas9
• Expression is robust in differentiated progeny including cardiomyocytes and neurons
• XPRESSO is accessible and does not require colony picking or proprietary equipment

## Graphical abstract

**Figure:**
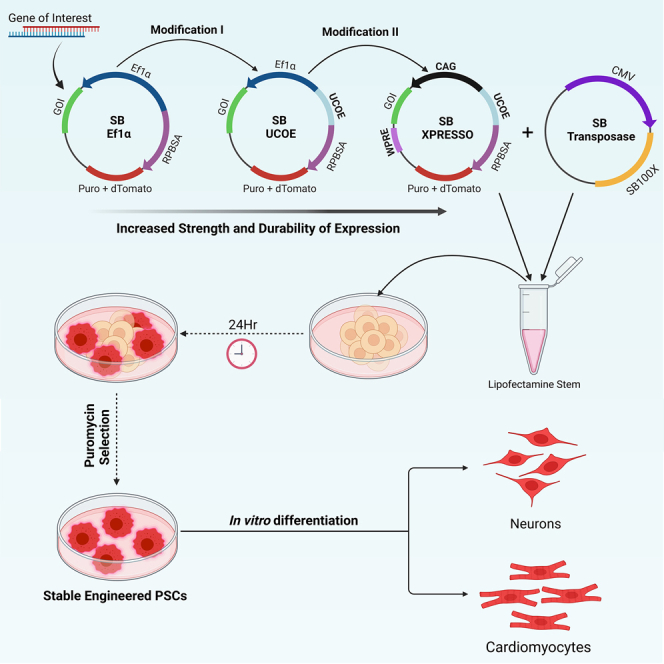

## Teaser

In this article, Wexler et al. introduce XPRESSO, a modular Sleeping Beauty transposon vector enabling rapid and stable genetic modification of hPSCs. By overcoming epigenetic silencing, XPRESSO ensures robust, transgene-independent expression in hPSCs and their progeny over time. This new tool expands the accessibility and reliability of genetic engineering in stem cell research.

## Introduction

Constitutive expression of ectopic proteins in human pluripotent stem cells (hPSCs) is desirable for numerous applications, including gene therapies (Filareto et al., 2013; Hanna et al., 2007), immune modulation (Deuse et al., 2019), and electrophysiological actuation (Steinbeck et al., 2015; Wexler et al., 2023), all of which necessitate strong and durable expression of the relevant genetic cassettes. Prevailing methods for the creation of stable, genetically modified hPSC lines are hindered by factors such as limited insert size (Bulcha et al., 2021; Maier et al., 2010), biosafety considerations, insufficient and inconsistent expression (Hoffmann et al., 2017; Pfaff et al., 2013), low efficiency of homology-directed repair (Yu et al., 2015), and protracted cell culturing (see Table S1 for a detailed comparison of existing methods). These issues have greatly hindered the potential and accessibility of this powerful methodology. Moreover, hPSCs are particularly prone to epigenetic silencing of transgenes (Bagley et al., 1998; Ellis, 2005; Müller-Kuller et al., 2015), with certain constructs, such as optogenetic actuators, displaying a greater tendency for rapid silencing than others (Klapper et al., 2017). Thus, even successful genetic modification and generation of hPSC lines does not guarantee sufficient expression over time, and multiple attempts using distinct methods are often necessary before a line with stable expression is created. This stability is especially crucial when lengthy culture times and differentiation protocols, such as with hPSC-derived neurons (Hussein et al., 2023), are necessary.

To address these issues, we sought to develop a simplified and efficient approach for creating overexpression lines in hPSCs guided by three fundamental principles: (1) the genetic engineering process should be accomplished easily and rapidly, within 1–2 weeks, with minimal expertise and without the need for colony picking; (2) no specialized equipment such as cell sorters or electroporators should be required, increasing the accessibility of the technology; (3) engineered cells should maintain strong and gene-independent expression of the desired construct over extended periods of culturing and differentiation.

In accordance with these principles, we sought a convenient delivery mechanism capable of inserting large genetic cassettes into the genome of hPSCs. Recently, the Sleeping Beauty (SB) transposon vector has shown great promise as a non-viral gene transfer method *in vitro* (Ammar et al., 2012) and is currently being tested in multiple gene therapy clinical trials (Kebriaei et al., 2017). Owing to the development of a novel hyperactive SB transposase (Mátés et al., 2009) and improvements in non-viral gene transfer reagents (Yamano et al., 2010), highly efficient genomic insertions of large (>100 kb) (Rostovskaya et al., 2012) elements across a wide array of cell types are now achievable using this technology. The SB transposon system is especially appealing due to its nearly random insertion profile at TA nucleotide pairs (Huang et al., 2010; Zhang et al., 2013). This is in stark contrast to Tol2 and PiggyBac transposons (Huang et al., 2010) as well as viral gene transfer methods (Schröder et al., 2002) that possess biases for insertion into open reading frames and transcriptional start sites. For these reasons, we opted to use a modular SB transposon for genomic insertion. We also determined to use a chemical transfection reagent for the gene transfer in accordance with our desire to make this technology accessible.

Here, we report the creation of XPRESSO (expedited persistent and robust engineering of stem cells with sleeping beauty for overexpression), a modified, anti-silencing, SB transposon vector coupled with a streamlined workflow that, together, allows for rapid generation of genetically modified hPSCs in a robust and repeatable manner. Using this technology, we demonstrate that hPSCs can be genetically modified for the overexpression of various proteins and small RNAs in 1–2 weeks without proprietary equipment or colony picking. We further show that expression is not silenced in undifferentiated hPSCs over long periods of time (>150 days) and that functional activity of the inserted proteins is maintained in both undifferentiated hPSCs as well as differentiated cells such as cardiomyocytes and neurons.

## Results

### Optimized genetic engineering of hPSCs using the SB transposon

**Figure 1:**
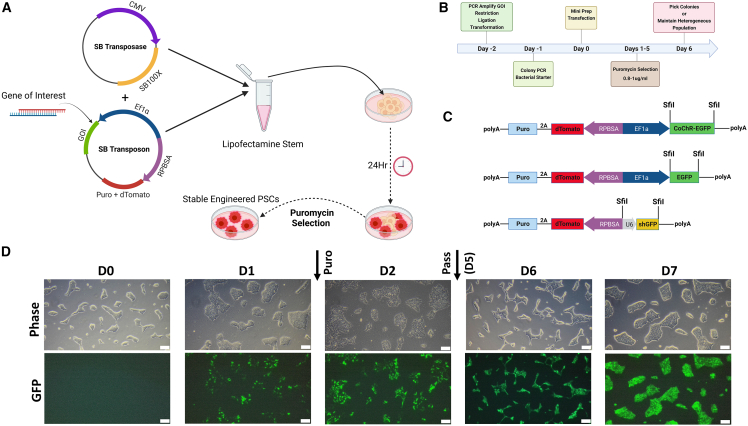
Genetically engineering hPSCs using the sleeping beauty transposon system (A) Schematic representation of Sleeping Beauty (SB) engineering of hiPSCs. Genes of interest (GOI) are cloned into the SB donor plasmid, which is co-transfected with the SB100X transposase plasmid into the hiPSCs. Antibiotic selection is started after 24 h and continued for 6–7 days. (B) Timeline of SB transfection method. On day 2, the GOI is cloned into the donor plasmid. On day 1, the plasmid with the GOI is amplified. On day 0, the amplified plasmid is purified and used to transfect the hiPSCs at 15%–40% confluence. On days 1–5, the hiPSCs are subjected to puromycin selection. By day 6, isolated colonies are clearly visible and can be dissected. Alternatively, a heterogeneous population of engineered hiPSCs can be maintained. (C) Graphical representation of the three GOI inserted into the SB donor vector using the SfiI restriction site. (D) Representative fluorescent and phase-contrast microscopy of hiPSCs throughout the process of generating a stable EGFP expressing line. The process begins on D0 when cells are transfected. On D1 (24 h post-transfection), puromycin is added to the feeding medium. At D5, the cells are passaged to create a polyclonal line. On D7, after passaging, once ∼100% of the transfected cells are GFP-positive and typical stem cell colonies are observed, selection is stopped. 4× objective; scale bars, 200 μm.

We began by developing an optimized workflow and delivery strategy for the SB transposon and transposase plasmids that would allow for efficient and rapid generation of genetically modified hPSC lines through chemical transfection (Figures 1A and 1B). Using a bicistronic SB transposon (Kowarz et al., 2015) and the hyperactive SB100X transposase (Mátés et al., 2009), we created three distinct proof-of-concept plasmids containing: (1) the fluorescent protein EGFP; (2) the optogenetic channel CoChR (Klapoetke et al., 2014) fused to EGFP; and (3) the GFP-targeting shRNA shGFP, with all plasmids co-expressing a puromycin resistance gene for selection (Figure 1C). The hPSCs were grown in a feeder-free culture and co-transfected with transposon and transposase plasmids. Puromycin selection was applied 24 h after transfection. A significant number of positive colonies were observed within 24 h of selection, and a ∼100% positive heterogeneous population, comprised of dozens of genetically unique clones, became confluent and ready for passaging within 4–7 days (Figure 1D). Following 7 days of selection, no transiently transfected, or non-transfected, cells remained (Figure S1A). The genetically modified hPSC lines were maintained as heterogeneous populations to minimize random and positional effects on the quantified stability and efficacy of this method.

### Functional evaluation of transgenes in hPSCs and derived cardiomyocytes

**Figure 2:**
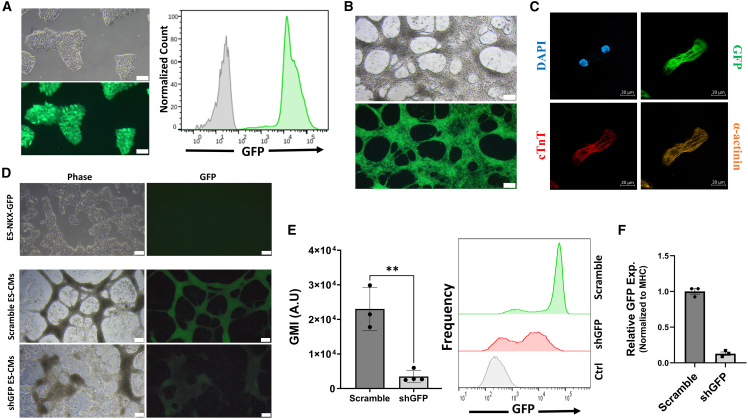
Functional expression of reporter and shRNA constructs in hPSCs and derived cardiomyocytes (A) Representative fluorescent and phase-contrast microscopy (left) and flow cytometry histogram (right) of P2 (D15) SB-EGFP-hiPSCs. Notice that ∼100% of the cells are GFP-positive. 10X lens; scale bars, 100 μm. (B) Representative fluorescent and phase-contrast microscopy of EGFP hiPSC-CMs 14 days after differentiation of P4 EGFP iPSCs. 4X lens; scale bars, 200 μm. (C) Immunostaining of EGFP-hiPSC-CMs for cTnT (red), α-actinin (orange), EGFP (green), and DAPI (blue). X63 magnification; scale bars, 10 μm. (D) Representative fluorescent and phase-contrast microscopy images of NKX2.5-GFP embryonic stem cells (top) and derived cardiomyocytes expressing scramble (middle) and shGFP (bottom) shRNAs. Note how the stem cells are GFP-negative because of the cardiac specific promoter and how GFP expression is significantly diminished in cardiomyocytes expressing shGFP. 4X lens; scale bars, 200 μm. (E) Flow cytometry GFP intensity histograms of NKX2.5-scramble and NKX2.5-shGFP hESC-CMs (*n* = 3 and 4 biological replicates respectively) on day 18 of differentiation and corresponding bar graphs. Data presented as mean ± SEM; ^∗∗^*p* < 0.01; unpaired Student’s t test. (F) Quantitative PCR measurement of GFP mRNA knockdown in NKX2.5-shGFP hESC-CMs compared with NKX-scramble hESC-CMs, both normalized to MHC expression (*n* = 3 biological replicates in each group). Data presented as mean ± SEM.

After establishing the genetically modified hPSC lines, puromycin selection was stopped, and the expression and functional activity of the transgenes were evaluated in undifferentiated hPSCs and differentiated cardiomyocytes (hPSC-CMs). The EGFP-expressing human-induced PSCs (hiPSCs) were created using two healthy control hiPSC lines. The generated lines showed strong fluorescence in ∼100% of cells, allowing for easy tracking and visualization (Figure 2A). Differentiated hiPSC-CMs retained EGFP expression and stained positive for cardiac-specific markers (Figures 2B, 2C, S1B, and S1C; Video S1).

Video S1. hiPSC-CMs differentiated from the Ef1a-EGFP line, related to Figure 2

To demonstrate the functionality of generating stable hPSC lines expressing shRNAs, which can facilitate consistent knockdown of proteins, we used a previously characterized (Protze et al., 2017) human embryonic stem cell (hESC) line expressing EGFP under the cardiac-specific promoter, NKX2.5 (Figure S2A). Both stable shGFP and scramble shRNA lines were generated. The fluorescent intensity of NKX2.5-driven EGFP was significantly diminished (∼6.6-fold) in cardiomyocytes derived from the shGFP line as compared to the scramble shRNA line, and significant knockdown (∼87%) of EGFP transcripts was observed (Figures 2D–2F), validating the correct expression and silencing efficacy of the inserted shRNA. No difference in GFP fluorescence was observed between the scramble shRNA line and parental NKX2.5 ES line (Figure S2B). Comparable results were observed when we created stable shGFP- and scramble-shRNA-expressing lines from healthy control hiPSCs. Following cardiomyocyte differentiation, the resulting hiPSC-CMs were transduced with AdV-GFP, and fluorescent intensity was quantified (Figure S2C), showing diminished EGFP expression in the shGFP-hiPSC-CMs.

**Figure 3:**
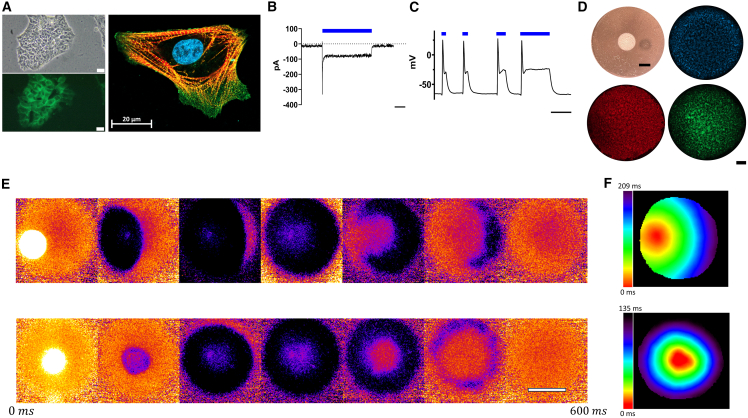
Optogenetic hiPSCs and applications in derived cardiomyocytes (A) Fluorescent (left-bottom) and phase-contrast (left-top) microscopy of SB-CoChR-hiPSCs on day 11 post-transfection and confocal microscopy of a differentiated CM (right). Membrane localization of the optogenetic channel CoChR (green) is clearly visible. Fluorescent microscopy using 40× objective, GFP filter, scale bars, 20 μm. Confocal microscopy 63× oil objective, DAPI: blue, EGFP: green, α-actinin: orange, cTnT: red. (B) Representative voltage-clamp recording of an optogenetic hiPSC-CM held at −60 mV and stimulated with 470 nM light. Blue bar indicates the time of stimulation. Scale bars, 1 s. (C) Representative current-clamp recording of an optogenetic hiPSC-CM stimulated with 470 nM light. Blue bar indicates the time of stimulation. Notice the ability to both stimulate AP generation as well as modulate its properties. Scale bars, 1 s. (D) Generation of the CoChR-expressing hiPSC-derived cardiac cell sheets (CCSs). Top left image is a macroscopic view of a CCS. Scale bars, 5 mm. Remaining images are representative immunofluorescent images (DAPI: blue, cTnT: red, EGFP: green). 5× objective, stitched image, scale bars, 1 mm. (E) Snapshots (every 100 ms) taken from a dynamic display, showing the optical mapping results of an optogenetically paced CCS at 1 Hz (top: paced from the left side; bottom: paced from the center). (F) Electrical activation maps of a CCS optogenetically paced from the left side (top) and the center (bottom). The red and purple colors represent the earliest and latest times of electrical activation, respectively.

We next created optogenetic CoChR-hiPSC lines using two distinct control hiPSC lines. The optogenetic channel, which was fused to the fluorescent protein EGFP, demonstrated robust membrane-localized expression in the undifferentiated hiPSCs as well as in differentiated cardiomyocytes (Figures 3A and S3A). Light sensitivity of the CoChR-hiPSC-CMs was observable within the differentiation plate in the form of altered contraction in response to illumination (Video S2). We next assessed the electrophysiological properties of the CoChR-hiPSC-CMs using patch-clamp recordings. Depolarizing optogenetic currents were observed during voltage-clamp recordings in response to 470 nm light with characteristic maximal and steady-state currents (Mattis et al., 2012) (Figure 3B). Current clamp recordings revealed the ability of short illumination pulses to induce action potentials (APs) in the CoChR-hiPSC-CMs, while longer pulses could also modulate AP duration (APD) (Figure 3C). To further demonstrate the utility of this optogenetic hiPSC line, we created a two-dimensional tissue model (Shaheen et al., 2018) based on confluent cardiac cell sheets (CCSs) from the CoChR-hiPSC-CMs (Figure 3D). The generated CCSs could be optogenetically paced at various frequencies and from different locations using focal illumination, resulting in the development of an activation wavefront propagating from the illumination site to activate the entire culture (Figures 3E and 3F; Video S3).

Video S2. hiPSC-CMs differentiated from the Ef1a-CoChR line, related to Figure 3

Video S3. hiPSC-CMs differentiated from XPRESSO-CoChR line, related to Figure 5

### Gene-specific epigenetic silencing and anti-silencing strategies

**Figure 4:**
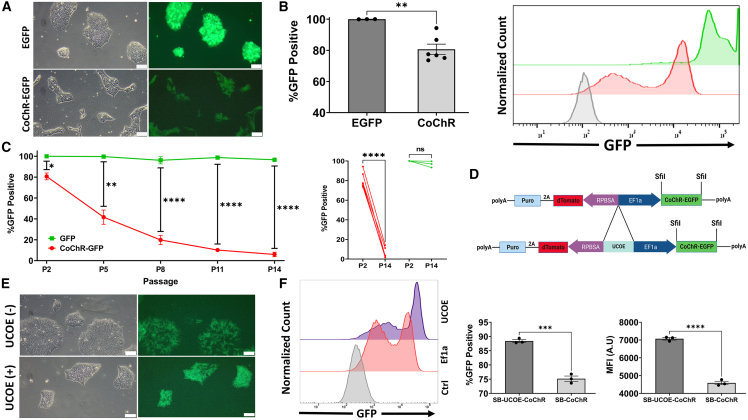
Expression of an optogenetic channel in hiPSCs is hindered by early variegation and rapid silencing, which can be ameliorated by addition of the UCOE element (A) Fluorescent (right) and phase-contrast (left) microscopy of SB-EGFP (top) and SB-CoChR–EGFP-hiPSCs (bottom) on day 15 post-transfection. Notice that transgene expression is weaker in the SB-CoChR line with significant variegation of expression. 10× objective, scale bars, 100 μm. (B) Bar graph (left) comparing the percentage of EGFP-positive cells in the SB-EGFP and SB-CoChR lines (*n* = 3 and *n* = 6 unique lines, respectively, each generated from two different control hiPSC lines) at P2 (D13), and representative offset histograms of SB-EGFP (green), SB-CoChR (red), and negative control (gray) hiPSCs showing markedly enhanced EGFP expression in SB-EGFP compared to SB-CoChR iPSCs. Mean ± SEM. Unpaired t test with Welch’s correction. ^∗∗^*p* < 0.01. (C) Percentage of EGFP-positive cells from SB-EGFP and SB-CoChR lines (*n* = 3 and *n* = 6 unique lines, respectively, each generated from two different control hiPSC lines) over 14 passages (∼60 days) (left) and paired comparison of P2 and P14 (right). Two-way ANOVA with the Geisser-Greenhouse correction and Šidák correction for multiple comparisons (left) and paired t test (right). ^∗^*p* < 0.05, ^∗∗^*p* < 0.01, ^∗∗∗∗^*p* < 0.0001. (D) Schematic diagram of the creation of the SB-UCOE-CoChR vector. (E) Fluorescent (right) and phase-contrast (left) microscopy of SB-CoChR (top) and SB-UCOE-CoChR (bottom) hiPSC lines 12 days after transfection. Notice that the expression of the GFP-linked optogenetic channel is markedly increased in the SB-UCOE-CoChR line with less variegation. 10× objective, scale bars, 100 μm. (F) Representative offset histograms (left) of SB-UCOE-CoChR (purple), SB-EF1α-CoChR (red), and negative control (gray) hiPSCs showing improved EGFP expression in SB-UCOE-CoChR compared to SB-EF1α-CoChR hiPSCs and bar graphs comparing the percentage of EGFP-positive cells (middle) and geometric mean intensity of EGFP fluorescence (right) in SB-EF1α-CoChR and SB-UCOE-CoChR hiPSC lines (*n* = 3 in both) at P2 (D13 after transfection). Mean ± SEM. Unpaired t test. ^∗∗∗^*p* < 0.001, ^∗∗∗∗^*p* < 0.0001.

While generating the above transgenic lines, we observed differential transgene expression between EGFP lines and optogenetic CoChR lines. Even at the earliest measurable time points, during and immediately after antibiotic selection, a marked difference in fluorescent intensity and considerable variegation of CoChR expression were noticeable (Figures 4A and 4B). This difference occurred despite the fact that the average copy number in both lines was similar (Figure S3B), indicating that an epigenetic phenomenon is likely responsible for this discrepancy. Furthermore, over extended periods of culturing, we found that the optogenetic line underwent rapid and almost complete epigenetic silencing even though the copy number remained relatively stable (Figures 4C and S3C). In contrast, the EGFP line retained stable expression during the same period (14 passages, ∼60 days after transfection).

In an attempt to prevent the gradual silencing of inserted transgenes, we utilized a minimal ubiquitous chromatin opening element (UCOE), which has been shown to protect linked heterologous constructs from methylation (Zhang et al., 2017), to create a modified SB-UCOE vector capable of limiting CpG-methylation-dependent epigenetic silencing (Figure 4D). The modified SB-UCOE-CoChR vector resulted in improved expression and lower levels of variegation compared to the unmodified vector, findings that were observable immediately after selection (Figures 4E and 4F). The stability of expression over time was also significantly enhanced, leading to a 340% increase in GFP-positive cells and a ∼79% increase in geometric mean fluorescent intensity at P11 (∼D45) (Figure S3D). Similar results were observed for the linked dTomato-PuroR gene (Figure S3E). Notably, silencing of the optogenetic transgene was also observed when using a piggyBac transposon (Figures S4A and S4B), and the anti-silencing effect afforded by the addition of the UCOE element was not observed when a randomized sequence control spacer of identical length was used instead (Figures S4C and S4D).

**Figure 5:**
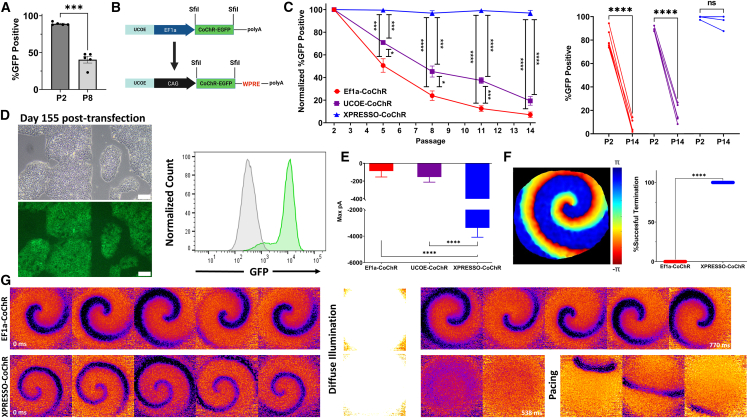
The novel XPRESSO vector prevents transgene silencing and significantly enhances expression in hiPSCs and derived cardiomyocytes (A) Bar graph comparing the percentage of EGFP-positive cells in SB-UCOE-CoChR lines (*n* = 5 unique lines generated from two different control lines) at P2 (∼D13) and P8 (∼D35). Mean ± SEM. Paired t test. ^∗∗∗^*p* < 0.001. (B) Schematic diagram depicting the creation of the XPRESSO-CoChR vector. (C) Change in the percentage of EGFP-positive cells (left) over 14 passages (∼55–60 days after transfection) from EF1α, UCOE, and XPRESSO hiPSC-CoChR lines (*n* = 6, 5, 4 distinct lines, respectively, generated in two different control hiPSC lines) normalized to the percentage of positive cells at the first measurement (P2) and paired comparison of EGFP expression in the different lines (right) from P2 (∼D13) and P14 (∼D57). Mean ± SEM. Mixed-effects analysis using the Geisser-Greenhouse correction and Tukey’s correction for multiple comparisons for the plots and paired t test for the paired comparison. ^∗^*p* < 0.05, ^∗∗∗^*p* < 0.001, ^∗∗∗∗^*p* < 0.0001. (D) Representative fluorescent (left-bottom) and phase-contrast microscopy (left-top) and normalized FACS histogram (right) of XPRESSO-CoChR hiPSCs on day 155 post-transfection. Approximately 94% of cells remained positive for EGFP expression. (E) Bar graph of the optogenetic current amplitudes measured in single-cell hiPSC-CMs (differentiated five passages [∼D24] after transfection). Engineered lines generated with the EF1α, UCOE, and XPRESSO CoChR vectors were compared (*n* = 5, 12, and 3 cells respectively). Significantly larger currents were observed in the XPRESSO-CoChR-hiPSC-CMs. One-way ANOVA with multiple comparisons using Tukey’s correction. ^∗∗∗∗^*p* < 0.0001. (F) Phase map of rotor-like arrhythmia generated in an optogenetic CCS (left) and bar graph (right) comparing the ability of optogenetic illumination to terminate rotor-like arrhythmias in EF1α-CoChR and XPRESSO-CoChR CCSs (*n* = 28 attempts from 5 CCSs and *n* = 47 from 10 CCSs, respectively). Fisher’s exact test. ^∗∗∗∗^*p* < 0.0001. (G) Snapshots every 77 ms taken from a dynamic display showing the optical mapping of EF1α-CoChR (top) and XPRESSO-CoChR (bottom) hiPSC-derived CCSs. Note how diffuse illumination has no effect on the rotor-like arrhythmia in the EF1α-CoChR CCS but easily terminates the arrhythmia in the XPRESSO-CoChR CCS and leads to a normal rhythm thereafter with electrical pacing (pacing frames 308 ms apart).

Even though the modified SB-UCOE-CoChR vector significantly slowed epigenetic silencing, by P8 (∼35 days after transfection), fewer than 50% of the engineered cells expressed detectable levels of the optogenetic channel (Figure 5A). Considering that many PSC differentiation protocols necessitate long culturing periods (D’Aiuto et al., 2014; Hogrebe et al., 2021; Hussein et al., 2023), we sought to improve the SB vector with the goal of achieving stable expression for at least 3 months (∼100 days). To this end, we designed the XPRESSO vector (Figure 5B) in which we kept the UCOE element, replaced the EF1α promoter with the CAG promoter, and added a WPRE element to increase nuclear export and transcript stability (Zufferey et al., 1999). The CAG promoter was chosen as it has been shown to be more refractory to silencing in PSCs (Hong et al., 2007), possibly due to the synthetic intron included in the promoter sequence (Seczynska et al., 2022). Lines generated using XPRESSO showed almost no observable epigenetic silencing over 25 passages (∼100 days) and had improved expression of the optogenetic channel compared to both the EF1α-CoChR and modified UCOE-CoChR SB vectors (Figures 5C, S5A, and S5B) in spite of a similar or lower average copy number (Figure S3B). This resulted in strong CoChR expression that was observed in hiPSCs >150 days after transfection and was maintained after differentiation for >45 days (Figures 5D, S5C, and S7A; Video S3). Functional experiments in differentiated hiPSC-CMs also demonstrated a marked increase in optogenetic current amplitudes as compared to cardiomyocytes derived from the lines generated with the EF1α-CoChR and UCOE-CoChR SB vectors (Figure 5E).

To highlight the translational potential of the increased optogenetic currents, we aimed to evaluate the ability to also silence cardiac electrical activity using optogenetics. Initially, we showed that spontaneously beating XPRESSO-CoChR-hiPSC-CMs could be continuously silenced by prolonged illumination (Video S3). Next, to test this functionality in a more clinically relevant model, we induced rotor-like arrhythmias (Figure 5F) in optogenetic CCSs that were generated using either the original EF1α-CoChR vector or the enhanced XPRESSO-CoChR vector. We observed that these arrhythmias could easily be terminated optogenetically in the XPRESSO-CoChR CCSs using diffuse light but were unaffected by the same illumination (in terms of amplitude and duration) in the EF1α-CoChR CCSs (Figures 5F and 5G; Video S3).

### The XPRESSO vector allows for strong and durable expression in hPSC-derived neurons

**Figure 6:**
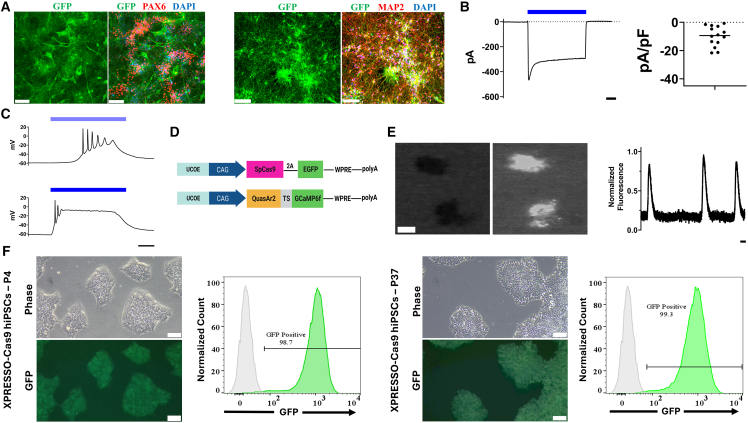
The XPRESSO vector enables the generation of optogenetic hiPSC-derived neurons and permits robust expression of versatile transgenes (A) Representative confocal microscopy of XPRESSO-CoChR neural progenitor cells (NPC) (left) and cortical neurons (right). EGFP: green, PAX6/MAP2 (NPC/cortical neuron): red, DAPI: blue. 20× objective, scale bars, 100 μm. (B) Representative voltage-clamp recording of an optogenetic hiPSC-derived neuron held at −60 mV and stimulated with 488 nM laser light. Blue bar indicates the time of stimulation. Scale bars, 1 s. (C) Representative current-clamp recording of spiking activity in an optogenetic hiPSC-derived neuron being stimulated with lower intensity (top) and higher intensity (bottom) 488 nM laser light. Notice how low-intensity light results in slightly delayed spiking activity, whereas the more intense light leads to immediate spiking followed by rapid silencing of electrical activity. Scale bars, 100 ms. (D) Graphical representation of the XPRESSO-Cas9 and XPRESSO-CaViar vectors. (E) Calcium imaging of XPRESSO-CaViar hiPSC-CM clusters within the differentiation plate (left image-pair); optical intracellular Ca^2+^ transients recorded from a single-cell XPRESSO-CaViar hiPSC-CM (right). Scale bars, 500 μm (left), 1 second (right). (F) Representative phase-contrast, fluorescent microscopy, and flow cytometry histograms of XPRESSO-Cas9 hiPSCs at passage 4 (left group) and at passage 37 after undergoing cryopreservation and thawing (right group) demonstrate that no significant silencing of Cas9 expression occurs over prolonged culturing.

To demonstrate the stability of expression in cells requiring prolonged differentiation procedures, we applied a ∼3-month cortical neuron differentiation protocol (Hussein et al., 2023) to the XPRESSO-CoChR line. The generated neural progenitors and terminally differentiated cortical neurons stained positive for their characteristic markers while displaying expression of the optogenetic channel (Figures 6A and S6). Patch-clamp experiments in terminally differentiated cortical neurons demonstrated the presence of robust optogenetic currents (Figure 6B), which led to significant membrane depolarization and neuronal spiking in response to 488 nm illumination (Figures 6B and 6C).

### The XPRESSO vector allows for the creation of hPSC lines expressing versatile transgenes

Finally, we created two additional SB constructs, XPRESSO-CaViar, containing a fusion fluorescent calcium and voltage indicator (Dempsey et al., 2016), and XPRESSO-Cas9, containing the SpCas9 protein (Ran et al., 2013). This was done to highlight the versatility of our method and create vectors that may be beneficial to the stem cell community (Figure 6D). Differentiated cardiomyocytes derived from the XPRESSO-CaViar hiPSC line displayed bright calcium-dependent fluorescence. This allowed for recordings of optical traces from both cardiomyocyte clusters within the differentiation plate as well as from dissociated single-cell hiPSC-CMs (Figure 6E; Video S4).

Video S4. hiPSC-CMs differentiated from XPRESSO-CaViar and XPRESSO-Cas9 lines, related to Figure 6

**Figure 7:**
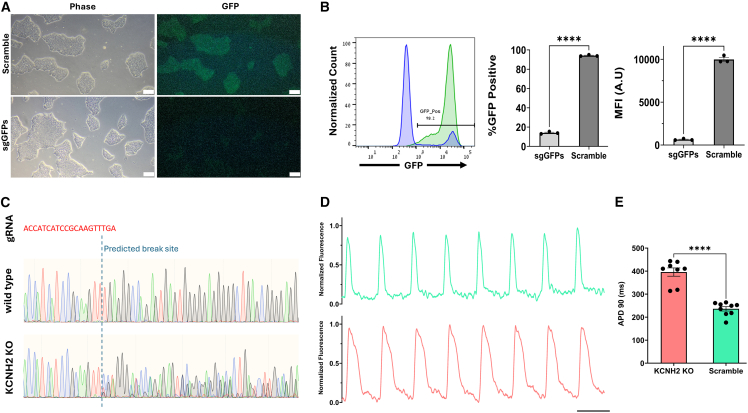
The XPRESSO-Cas9 hiPSC line permits high-efficiency knockout of genes without selection (A) Representative phase-contrast (left) and fluorescent microscopy (left) of Cas9 expressing hiPSCs 6 days after transfection with either scramble (top) or sgGFP (bottom) guide RNA. 4× objective, scale bars, 200 μm. (B) Representative flow cytometry histograms (left) comparing sgGFP (blue) and scramble (green) transfected XPRESSO-Cas9 hiPSCs and the corresponding bar graphs comparing the percentage of EGFP-positive cells (middle) and the geometric mean fluorescent intensity (right) 6 days after transfection (*n* = 3 biological replicates in each group). Data presented as mean ± SEM. ^∗∗∗∗^*p* < 0.0001, unpaired Student’s t test. (C) Sequencing of the KCNH2 gene demonstrates that XPRESSO-Cas9 hiPSCs transfected with sgRNA targeting KCNH2 show significant indels downstream of the gRNA. Non-transfected hiPSCs from the same line show no abnormalities at the same locus. (D) Representative optical mapping traces from CCSs derived from the hiPSC XPRESSO-Cas9 line following transfection with scramble (top) or KCNH2-targetting sgRNA (bottom). Marked elongation of the action potential duration (APD) can be seen in the KCNH2 KO line. Scale bars, 1 s. (E) Bar graphs comparing the APD measured at 90% of repolarization (APD90) in cardiac cell sheets derived from hiPSCs transfected with either scramble or KCNH2-targeting sgRNA (*n* = 9 and *n* = 8 CCSs, respectively, from two different differentiations). Data presented as mean ± SEM. ^∗∗∗∗^*p* < 0.0001, unpaired Student’s t test.

When generating the XPRESSO-Cas9 hiPSCs, a single colony was picked in order to create a monoclonal line. This was done to increase the reproducibility and consistency of knockouts (KOs) after gRNA transfection. The established XPRESSO-Cas9 line was shown to constitutively express the Cas9 enzyme and the fluorescent reporter EGFP in hiPSCs and differentiated cardiomyocytes. This expression was not silenced over time (>120 days) or following differentiation (Figures 6F and S7B; Video S4). To assess the functionality and KO efficiency of this line, we co-transfected the undifferentiated hiPSCs with either EGFP-targeting sgRNAs or a scramble guide RNA (gRNA). We then measured the expression of EGFP after 6 days using flow cytometry and noted highly efficient KO of EGFP without antibiotic selection or cell sorting. A ∼94% decrease in geometric mean fluorescent intensity was observed in cells transfected with single-guide GFPs (sgGFPs) compared to cells transfected with scramble gRNAs, and ∼85% of the cells were EGFP negative, indicating successful KO in a majority of cells (Figures 7A and 7B). Lastly, to demonstrate the utility of this cell line, we targeted the endogenous KCNH2 gene for KO. The KCNH2 gene codes for the alpha subunit of the Kv11.1-voltage-gated potassium channel that mediates the late repolarizing I_K__r_ current in CMs and is implicated in long qt syndrome type II (LQT2). XPRESSO-Cas9 hiPSCs were transfected with either scramble or KCNH2-targetting sgRNAs, and sequencing results confirmed gene disruption (Figure 7C). Next, control and KO XPRESSO-Cas9 hiPSCs were differentiated into CMs and used to generate CCSs. The KO CCSs exhibited a significant prolongation of action potential duration at 90% repolarization (APD90) compared to control CCSs (395.93 ± 18.03 vs. 235 ± 9.57 ms, respectively; *n* = 8 and *n* = 9 CCSs from two separate differentiations; *p* < 0.0001), indicating successful KCNH2 disruption and recapitulation of the LQT2 phenotype (Figures 7D and 7E).

## Discussion

The genetic “toolbox” available to scientists has grown rapidly in recent years. Technologies such as genetically encoded indicators of pH (Rossano et al., 2013), voltage (Bando et al., 2019), calcium (Mank and Griesbeck, 2008), and cAMP (Zaccolo et al., 2000), as well as organelle and protein trackers (Enterina et al., 2015), have become indispensable research tools. At the same time, immunomodulatory proteins (Zhao et al., 2020), therapeutic peptides and genes (Wang et al., 2022), short RNAs (Zhu et al., 2022), and chimeric antigen receptors (Subklewe et al., 2019) hold immense therapeutic potential. Here, we set out to develop a methodology that would allow widespread implementation and utilization of these tools in hPSCs, and cells derived therefrom, by making the generation of genetically modified hPSC lines simple, cost-effective, and accessible.

To this end, we report the creation of XPRESSO, a novel “anti-silencing” SB vector created by cloning an epigenetic modifier, a post-transcriptionally active element, and a high expression constitutive promoter into a modular SB transposon plasmid. This novel vector enables near random insertion (Huang et al., 2010; Zhang et al., 2013) of large (Rostovskaya et al., 2012) genetic elements, resulting in robust expression and excellent stability over time. We have combined this vector with optimized molecular and cell culture techniques so that the overall efficiency achieved allows for simultaneous generation of multiple lines with minimal effort and resources. Notably, in this approach, only the transposon plasmid has been modified. This enables the use of engineered SB transposases with unique capabilities such as superior insertion profiles that favor introns and intergenic regions (Miskey et al., 2022), excisional activity allowing for the removal of inserted cassettes (Kesselring et al., 2020), and the use of RNA guides for targeted insertion (Kovač et al., 2020), all of which can be used in tandem with the XPRESSO vectors developed herein.

To demonstrate the broad applicability of this method, we have generated over 30 distinct genetically modified hPSC lines using six separate genes that were inserted into the different transposon vectors described herein. We also validated the functionality of the inserted genetic cassettes in undifferentiated hiPSCs and hESCs, as well as in differentiated cardiomyocytes and neurons.

Our results demonstrate a striking phenomenon of insert- and cell-type-specific epigenetic silencing. The addition of the ∼850 bp optogenetic CoChR channel upstream of the EGFP protein was sufficient to drastically change the expression pattern of the transgene over time, leading to almost complete loss of expression after only 14 passages. Interestingly, the same epigenetic silencing was not observed in HEK293 cells (data not presented), further emphasizing the importance of cell-type-specific methodologies for genetic engineering. Epigenetic silencing has been shown to be affected by the length of the insert as well as the specific sequence (Klapper et al., 2017; Seczynska et al., 2022). It is likely that both these elements play a role in determining which genes are silenced in hPSCs and to what degree. Importantly, our results highlight how crucial it is to assess multiple genes of different lengths when developing new methods and anti-silencing strategies for genetically engineering cells. Classic reporter genes, such as EGFP and other fluorescent proteins, appear to be expressed more robustly and silenced at a slower rate, if at all, potentially giving the false impression that the method used will garner similar results with longer or more complex proteins.

We chose to use an optogenetic actuator to illustrate the utility and versatility of this method, as optogenetic channels are powerful tools for investigating and modulating excitable cells and have been shown to be refractory to stable expression in hPSCs (Klapper et al., 2017). In agreement with these previous reports, we observed that expression of an optogenetic channel in undifferentiated hiPSCs is rapidly silenced. This limitation has impacted hPSC-derived cardiomyocyte and neuronal research, forcing scientists to rely on alternative approaches such as the use of tandem cell units (Jia et al., 2011), which are less physiological, or on the transduction of terminally differentiated cells, which leads to greater variability, limited throughput, and increased costs. Here, we show that using XPRESSO, long-term functional expression of an optogenetic channel can be achieved in hPSCs and their differentiated neurons and cardiomyocytes. Proof-of-concept studies then demonstrated the ability to robustly modulate the electrical activity of the optogenetic hiPSC-derived neurons and cardiomyocytes, so as to augment or suppress action potential (AP) generation in these cells. Moreover, in the case of the hiPSC-CMs, more complex interventions were demonstrated such as the ability to modulate APD and, in a hiPSC-based cardiac tissue model, the feasibility of optogenetic “pacing” and “defibrillation.”

This new methodological approach can also be used to easily insert larger transgenes, such as Cas9-EGFP (total integrated size >11 kb). Our results with this line indicate that constitutive Cas9 expression permits highly efficient genetic knockouts in hiPSCs without selection or cell sorting following a single chemical transfection of gRNAs. This is highly desirable for high-throughput CRISPR-based screens and is particularly useful in experiments using terminally differentiated cells that cannot divide and are therefore not amenable to antibiotic selection or cell sorting.

Throughout this project, we created several SB constructs containing genes that we believe will be of great utility to basic stem cell research, disease modeling, and drug screening. These include a genetically encoded combined voltage and calcium indicator, an optogenetic channel, and the cas9 enzyme. These plasmids will be part of a growing collection of XPRESSO vectors that we hope will serve as a valuable resource for the stem cell community.

It is important to note that this method is not a perfect fit for all genetic engineering applications. Knockouts, targeted gene corrections or base-pair edits, and endogenous protein tagging are all examples for which CRISPR-based techniques are more appropriate. However, as illustrated above, many genetic engineering applications simply require strong overexpression, for which random insertion is adequate or even preferable. The ability to achieve higher copy numbers and insert very large genetic cassettes are additional advantages of this method. Furthermore, stable Cas9-expressing lines, such as the one created in this report, can be used to increase the efficiency and ease with which targeted modifications can be achieved, illustrating how this method can be used in tandem with, rather than in place of, CRISPR-based methods.

A limitation of this technology is that its functionality is contingent on the constitutive CAG promoter, which elicits powerful expression of the inserted gene in undifferentiated stem cells and differentiated cells. There are instances in which expression of certain genetic cassettes would be undesirable or even toxic in stem cells or cases in which it is necessary to express the gene only in a specific subset of differentiated cells (e.g., cortical neurons, atrial cardiomyocytes). For these applications, tissue-specific or inducible promoters are often required. Further elucidation of the precise mechanisms underlying epigenetic silencing in hPSCs and differentiated cells may be instrumental to the development of a second-generation XPRESSO vector that can function irrespective of the upstream promoter. Also, as the CAG and WPRE elements were assessed together, the anti-silencing contribution of the WPRE and CAG elements alone were not quantified. It is likely that the effect the WPRE element has on transgene expression (Zufferey et al., 1999) contributes to the robust expression observed with the XPRESSO vector. Recent reports have demonstrated that the WPRE element is critical for anti-silencing (Uenaka et al., 2025), in addition to the necessity of the CAG promoter (Blanch-Asensio et al., 2024).

An additional limitation of this study is that we did not generate an EF1α-Cas9-EGFP line and, thus, could not determine if KO efficiency was improved comparatively in the XPRESSO-Cas9 line. Though other reports have shown similar proteins are heavily silenced in hiPSCs (Karbassi et al., 2024), indicating the XPRESSO vector may be crucial here, future work comparing the KO efficiency of an endogenous gene using polyclonal lines using these two vectors may further highlight the improved efficacy of the XPRESSO vector. Due to the nature of pooled genetically engineered hiPSC lines, we did not directly measure the methylation patterns at the SB transposon integration sites. Future studies using many clonal lines and next-generation methylation sequencing techniques might improve our understanding of the epigenetic silencing process we observed.

In conclusion, the methodology developed in this report accomplishes the three primary design goals we set out to achieve, namely, the ability to produce genetically modified hPSC lines in ∼10 days with minimal expertise, which maintain robust and stable expression over time, without necessitating colony picking or the use of proprietary equipment. We believe that this method will enable scientists from varying fields and backgrounds to incorporate genetically modified hPSCs into their research, significantly expanding the accessibility and impact of this important technology.

## Methods

Detailed methods can be found in the supplemental information. Key methods are described in brief below.

### Propagation and cardiomyocyte differentiation of hPSCs

Undifferentiated stem cells were grown using mTeSR1 medium (Stemcell Technologies) and passaged twice weekly. For differentiation of PSCs to cardiomyocytes, lines from passage 2–10 were used unless otherwise stated, except for the Ef1α-CoChR line in which passages 2–5 were used. A modified monolayer-directed differentiation protocol was used as previously described (Shinnawi et al., 2015). All stem cells produced from healthy controls were approved by the IRB (Helsinki) committee of Rambam Medical Center.

### Cortical neuronal progenitor cell differentiation

CoCHR-GFP-WPRE hiPSCs were cultured in mTeSR plus (Stemcell Technologies) in Matrigel-coated 6-well plates (Corning, cat# 07-200-83). Cortical NPCs were generated as described (Hussein et al., 2023).

### Plasmid construction, propagation, and harvesting

The original SB transposon plasmid pSBbi-RP was a gift from Eric Kowarz (Kowarz et al., 2015) (Addgene plasmid #60513), and the SB100X transposase plasmid pCMV(CAT)T7-SB100 was a gift from Zsuzsanna Izsvak (Mátés et al., 2009) (Addgene plasmid #34879). To generate the UCOE-SB plasmid backbone, we synthesized a minimal UCOE element (Synbio Technologies) previously described (Zhang et al., 2017) and used restriction enzyme cloning to insert it upstream of the EF1α promoter. To generate the XPRESSO vector, we used the modified UCOE-SB vector backbone and restriction cloning to remove the EF1α promoter and insert the CAG promoter, which was excised from the pCAGIG plasmid, a kind gift from Connie Cepko (Matsuda and Cepko, 2004) (Addgene plasmid #11159). Finally, we inserted the WPRE element downstream of the stop codon using restriction cloning. The CoChR transgene was kindly provided by Ofer Yizhar (Weizmann Institute). The CaViar gene was amplified from the pJMK074: CMV QuasAr2-TS-GCaMP6f plasmid, which was a gift from Adam Cohen (Dempsey et al., 2016) (Addgene plasmid #72303). The Cas9-T2A-EGFP gene was amplified from pSpCas9(BB)-2A-GFP (PX458), which was a gift from Feng Zhang (Ran et al., 2013) (Addgene plasmid #48138). A simplified protocol for the design of primers with adapter sequences followed by cloning instructions can be found in Note S1.

### Transfection and antibiotic selection

Using a modified version of a previously reported protocol (Giacalone et al., 2018), transfection wells were aspirated 30 min before transfection, and 1.5 mL of mTeSR1 medium was added. The transposon and transposase plasmids at a 3:1 molar ratio were mixed so that a total of 2 μg of plasmid DNA was used. The DNA was added to 50 μL of Opti-MEM (Gibco) and vortexed. Next, 12.5 μL of Lipofectamine Stem (Thermo Fisher Scientific) was added to the DNA, and the mixture was incubated at room temperature for 10–15 min. The mixture was added dropwise to each well. Puromycin (0.8–1 μg/mL) was added to the cells 24 h after transfection, upon observation of fluorescence. Selection medium was replaced daily, besides on the weekends. Once positive colonies were easily distinguishable (day 4–7 after transfection), either the entire well was disassociated and replated to create a heterogeneous genetically modified population or single colonies were picked to create clonal lines. Cells were then grown in selection medium for an additional 24 to 48 h and grown in mTeSR1 medium without puromycin from then on.

### Flow cytometry and analysis

Flow cytometry was performed on live and fixed cells using a BD LSR Fortessa II cytometer. Analysis was performed using FlowJo Software (BD Life Sciences). The gating strategy can be seen in Figure S7.

### gRNA transfection

In a 12-well plate, XPRESSO-Cas9 hiPSCs were transfected at ∼20%–40% confluence with two sgRNAs targeting EGFP/KCNH2 or with negative control scramble. Six days after transfection, EGFP expression was assessed using flow cytometry. For the KCNH2 KO experiment, XPRESSO-Cas9 hiPSC-CMs were disassociated and replated in 12-well plates at 85%–100% confluence and transfected in the same manner with KCNH2-targeting sgRNAs or scramble gRNA.

## Resource availability

### Lead contact

Further information and requests for resources and reagents should be directed to and will be fulfilled by the lead contact, Lior Gepstein (mdlior@technion.ac.il).

### Materials availability

We have deposited the empty XPRESSO vector, as well as vectors containing the inserts used in this study to Addgene plasmid repository (IDs 237295, 237296, 237297, 237298, and 237299). Plasmid maps and full plasmid sequence data are available there.

### Data availability

All datasets supporting the findings of this study are available from the corresponding author upon reasonable request.

## Acknowledgments

We thank Fang Zhang from the Thrasher lab for providing us with the sequence for the minimal 455bp UCOE element. We thank A. Elefanty and E. Stanley (Monash University, Victoria, AU) for providing the HES3-NKX2-5gfp/w reporter cell line.

Funding: this study was funded by the 10.13039/501100000781European Research Council (ERC-2017-COG-773181-iPS-ChOp-AF), by the Israel Science Foundation (grant no. 2001/23), and by the Zuckerman STEM Leadership Program.

## Author contributions

Conceptualization, Y.W., I.H., and L.G.; formal analysis, Y.W., H.G., I.R., and A.C.; investigation, Y.W., I.H., S.G., M.G., M.L., H.G., O.E., D.S., G.A., I.R., and A.C.; writing—original draft, Y.W.; writing—review & editing, Y.W., S.S., and L.G.; supervision, S.S. and L.G.

## Declaration of interests

The authors declare no competing interests.

## Supplemental information

Document S1. Figures S1–S7, Tables S1 and S2, Note S1, and supplemental methods

Document S2. Article plus supplemental information
