# Supplementary material for: XPRESSO: Rapid genetic engineering of human pluripotent stem cells for durable overexpression using a modular anti-silencing vector
Source: Stem Cell Reports. 2025 Aug 21;20(10):102603. doi: 10.1016/j.stemcr.2025.102603 (PMC12790730; doi:10.1016/j.stemcr.2025.102603)
Supplement: Document S1. Figures S1–S7, Tables S1 and S2, Note S1, and supplemental methods [file mmc1.pdf]

Stem Cell Reports, Volume *20*

Supplemental Information

XPRESSO: Rapid genetic engineering of human pluripotent stem cells for durable overexpression using a modular anti-silencing vector

Yehuda Wexler, Harel Grinstein, Irit Huber, Shany Glatstein, Matteo Ghiringhelli, Oded Edri, Michal Landesberg, Daniel Shiff, Gil Arbel, Idan Rosh, Ashwani Choudhary, Shani Stern, and Lior Gepstein

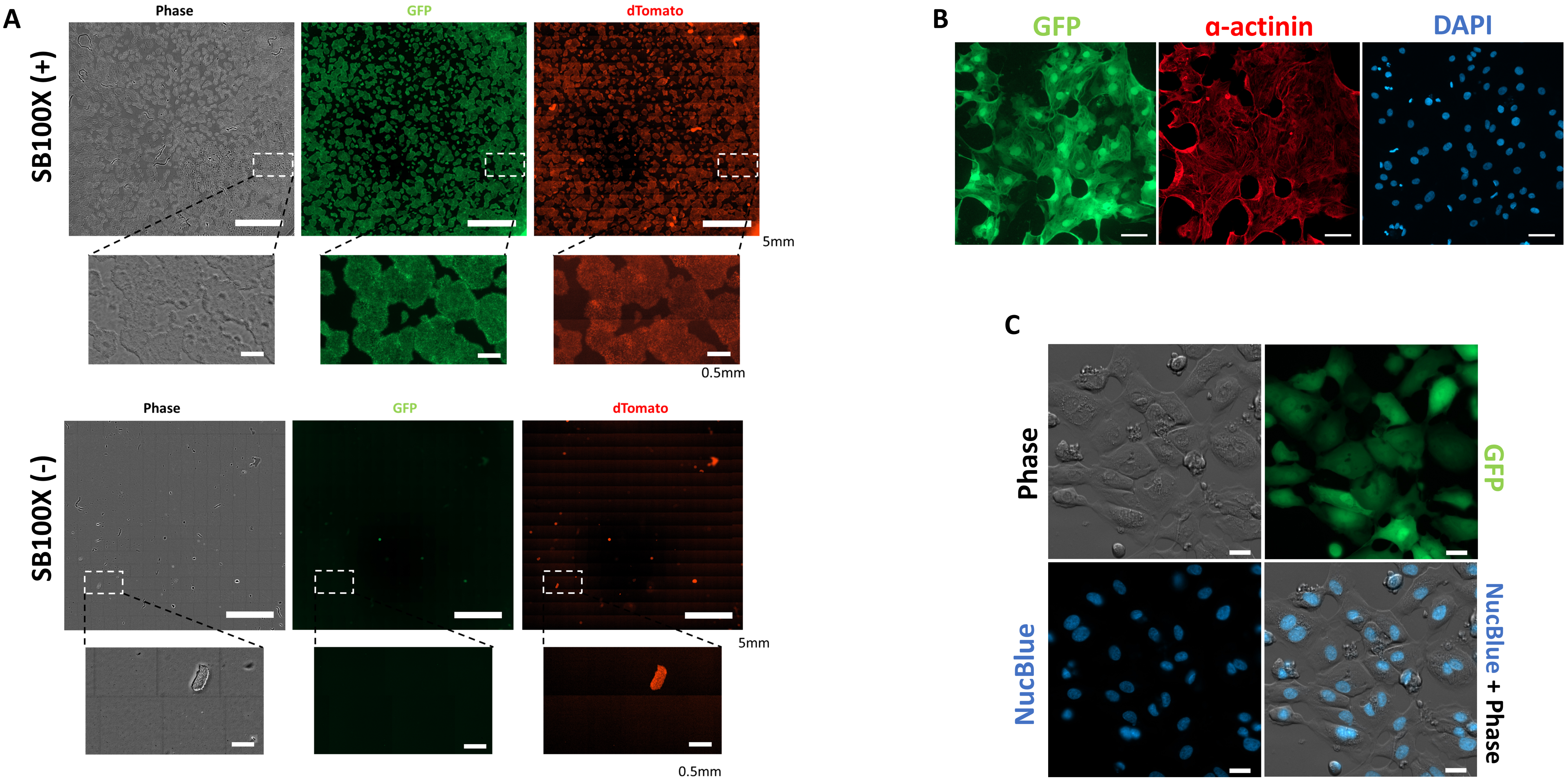

Figure S1. A. Images comparing transfection of a sleeping beauty plasmid (XPRESSO-CoChR-eGFP) with and without the SB100X transposase after 10 days (7 days of selection followed by 3 days without selection). As can be seen, in the SB100X (-) well, there are almost no colonies, and those that persist appear to be the result of random integration of the puromycin resistance gene without the CoChR-eGFP gene. 5x objective, image of the entire well stitched together. Scale-bars: 5 mm and 0.5 mm. **B**. Representative immunofluorescent images of Ef1a-eGFP hiPSC-CMs. 20x objective, scale-bar: 50 µm. **C**. Live cell imaging of Ef1a-eGFP hiPSC-CMs with nuclear staining using NucBlue. 20x objective, scale-bar: 50 µm.

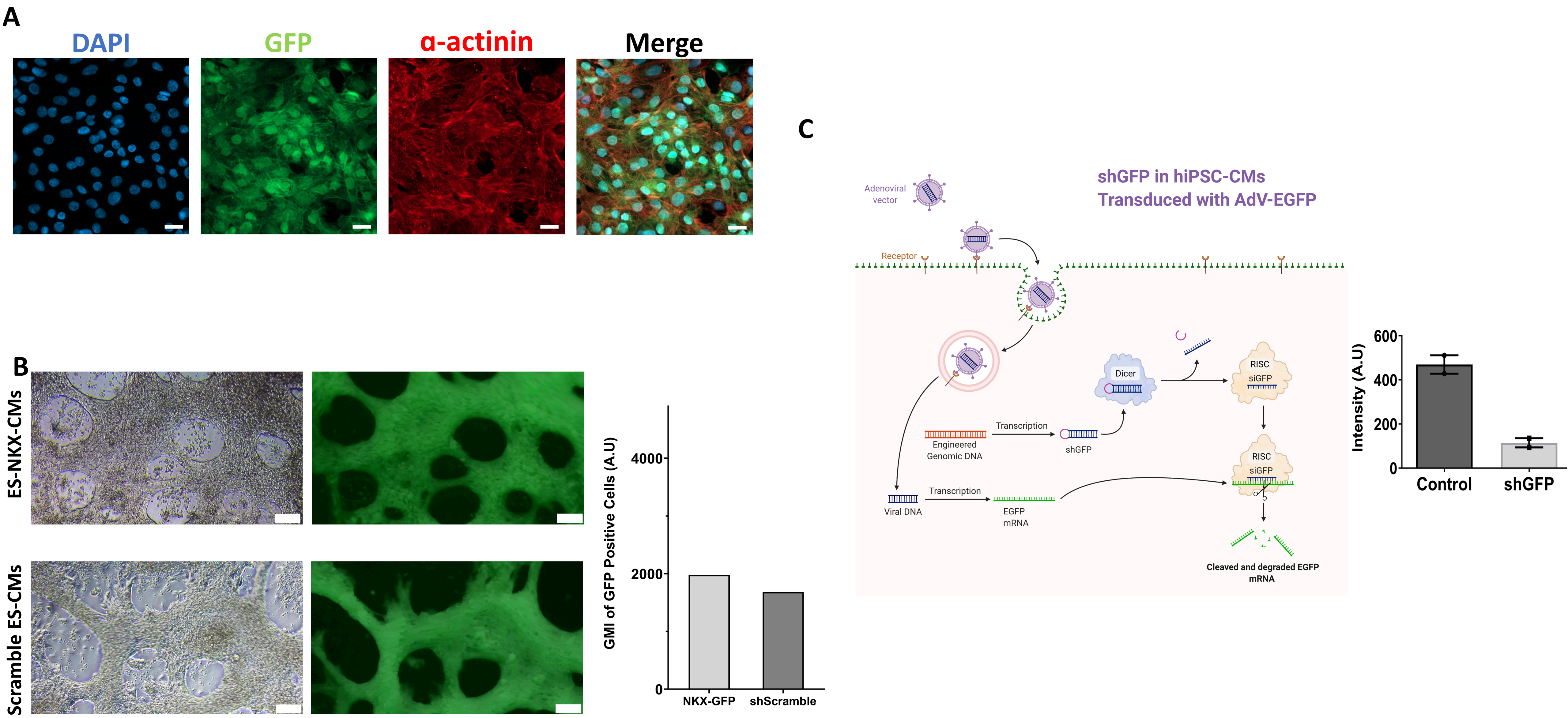

Figure S2. **A.** Immunofluorescent images of cardiomyocytes differentiated from the NKX2.5-eGFP ES line. Robust expression of the cardiac marker α-actinin and GFP can be seen. X20 objective, scale-bar: 20 μm. **B.** Representative fluorescent and phase-contrast microscopy images (left) of control (top) and scramble-shRNA expressing (bottom) NKX2.5-GFP ES-CMs, and bar-graph (right) of geometric mean fluorescent intensity (GMI) of GFP positive cells in parental NKX-GFP line and engineered scramble-shRNA line demonstrating similar fluorescent intensity. 10X objective, scale-bar: 100 μm. **C.** Schematic overview of adenoviral transduction of hiPSC-CMs genetically engineered to express shGFP (left) and bar graph comparing geometric mean fluorescent intensity between hiPSC-CMs expressing shGFP or scramble shRNAs six days after transduction with AdV-EGFP (right).

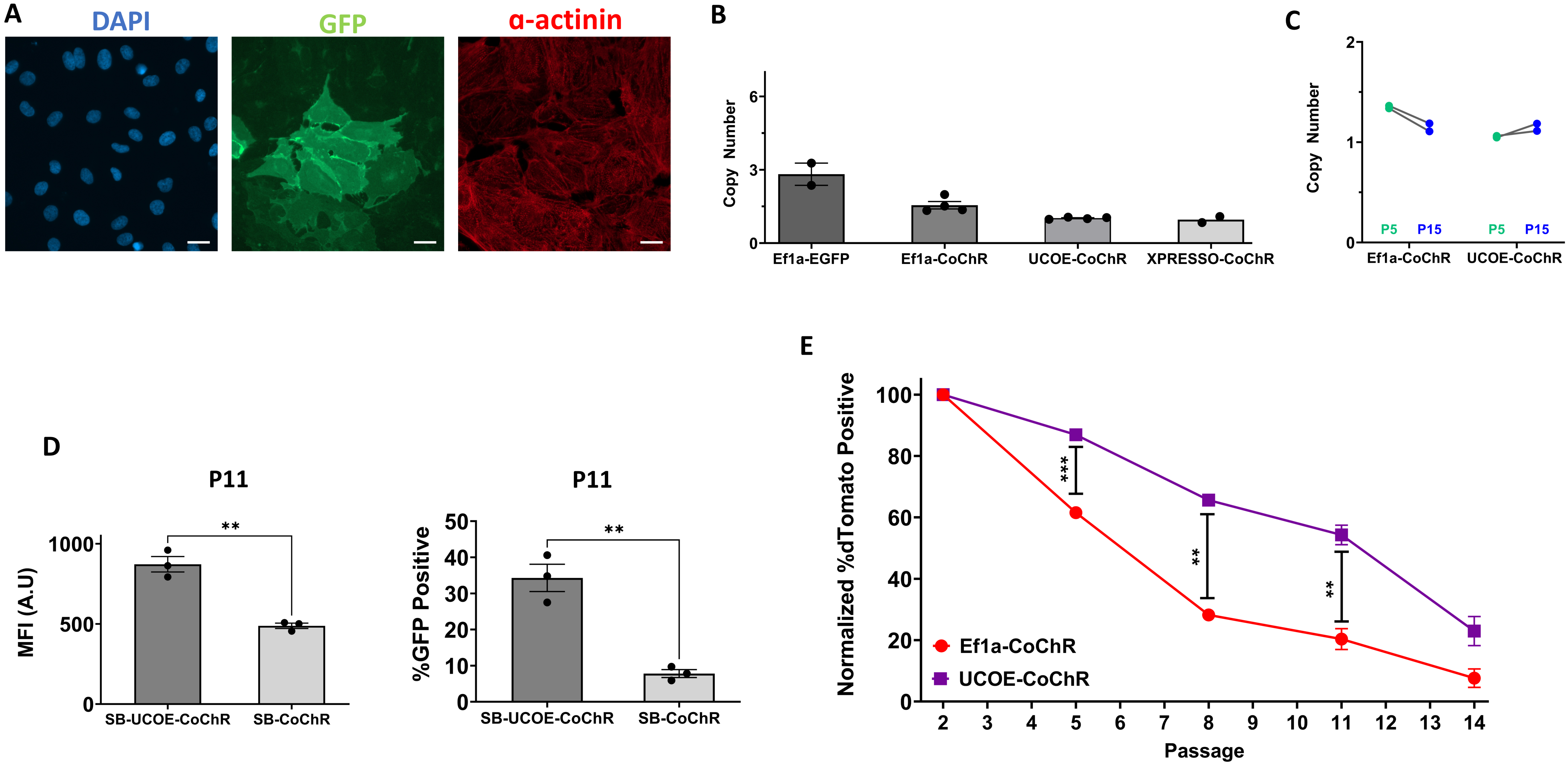

Figure S3. **A.** Immunofluorescent microscopy of an Ef1a-CoChR-eGFP hiPSC-CM cluster. 20x objective, scale-bar: 20 µm. **B.** Comparison of insertional copy number between Ef1a-EGFP, Ef1a-CoChR, UCOE-CoChR, and XPRESSO-CoChR lines. **C.** Paired comparison of insertional copy number at passages 5 (P5) and 15 (P15) in Ef1a-CoChR and UCOE-CoChR lines. **D.** Bar graphs comparing geometric mean fluorescent intensity and percentage of GFP positive cells between P11 hiPSC lines created with the original Ef1a-CoChR (n=3) or improved UCOE-CoChR (n=3) vectors. Student's t-test, $^{**}p<0.01$. **E.** Normalized percentage of dTomato positive cells from Ef1a-CoChR and UCOE-CoChR lines (n=3 for each) over 14 passages. Two-way ANOVA with the Geisser-Greenhouse correction and Šidák correction for multiple comparisons and individual variances. $^{***}p<0.001$ $^{**}p<0.01$.

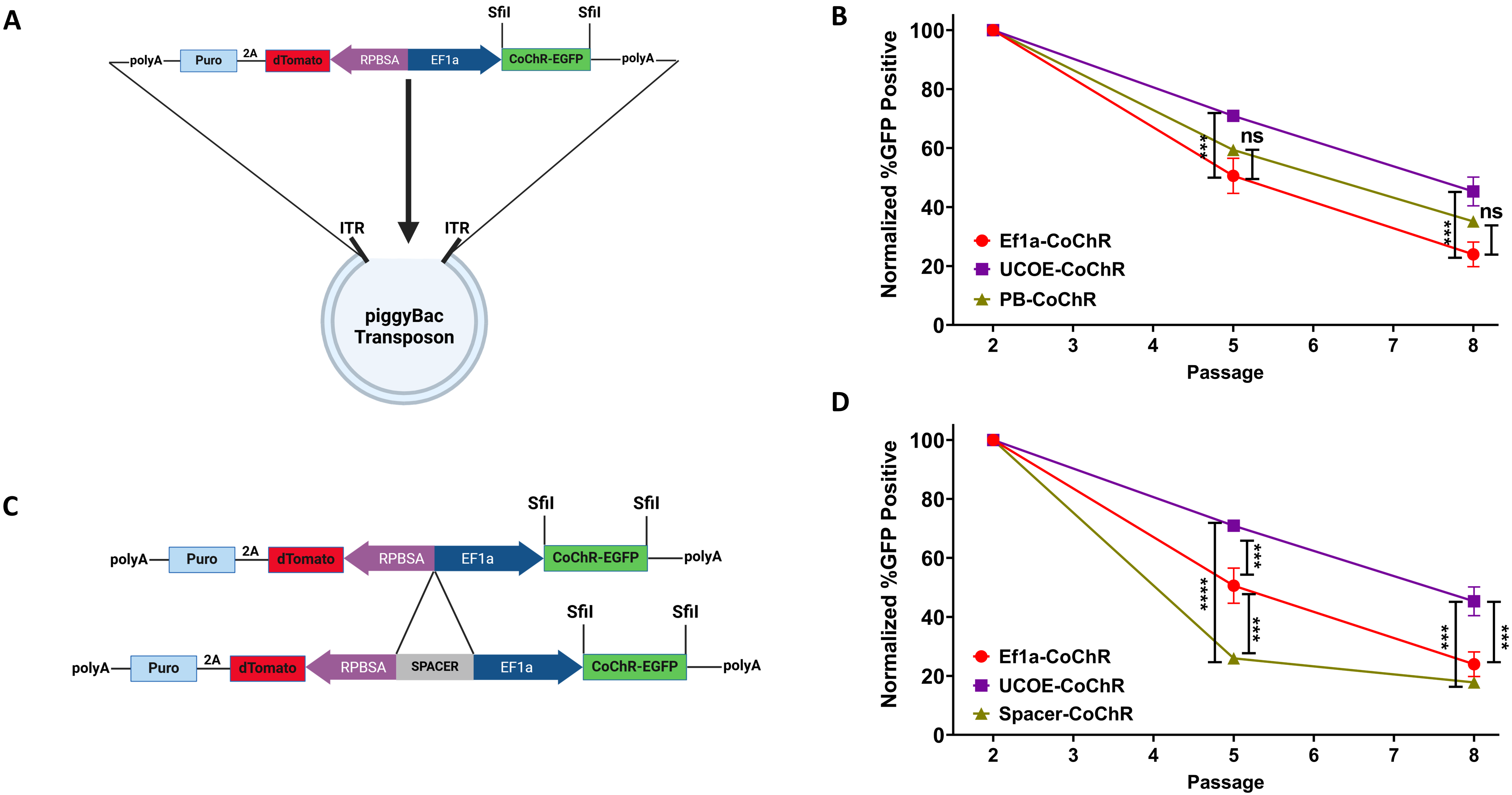

Figure S4. A. Schematic diagram of replacing the inserted construct from the original SB vector into a piggyBac transposon vector. **B.** Change in the percentage of eGFP positive cells over 8 passages (~35 days after transfection) from SB-EF1α, SB-UCOE, and PB-Ef1α hiPSC-CoChR lines (n=6,5,3 respectively) normalized to the percentage of positive cells at the first measurement (P2) **C**. Schematic diagram of inserting a random sequence control spacer between the Ef1a and RPBSA promoters. **D**. . Change in the percentage of eGFP positive cells over 8 passages (~35 days after transfection) from EF1α, UCOE, and Spacer hiPSC-CoChR lines (n=6,5,3 respectively) normalized to the percentage of positive cells at the first measurement (P2). Mean ± SEM. Two-way ANOVA using Tukey's correction for multiple comparisons and individual variances for each comparison. ***p<0.001, ****p<0.0001, ns- p>0.05.

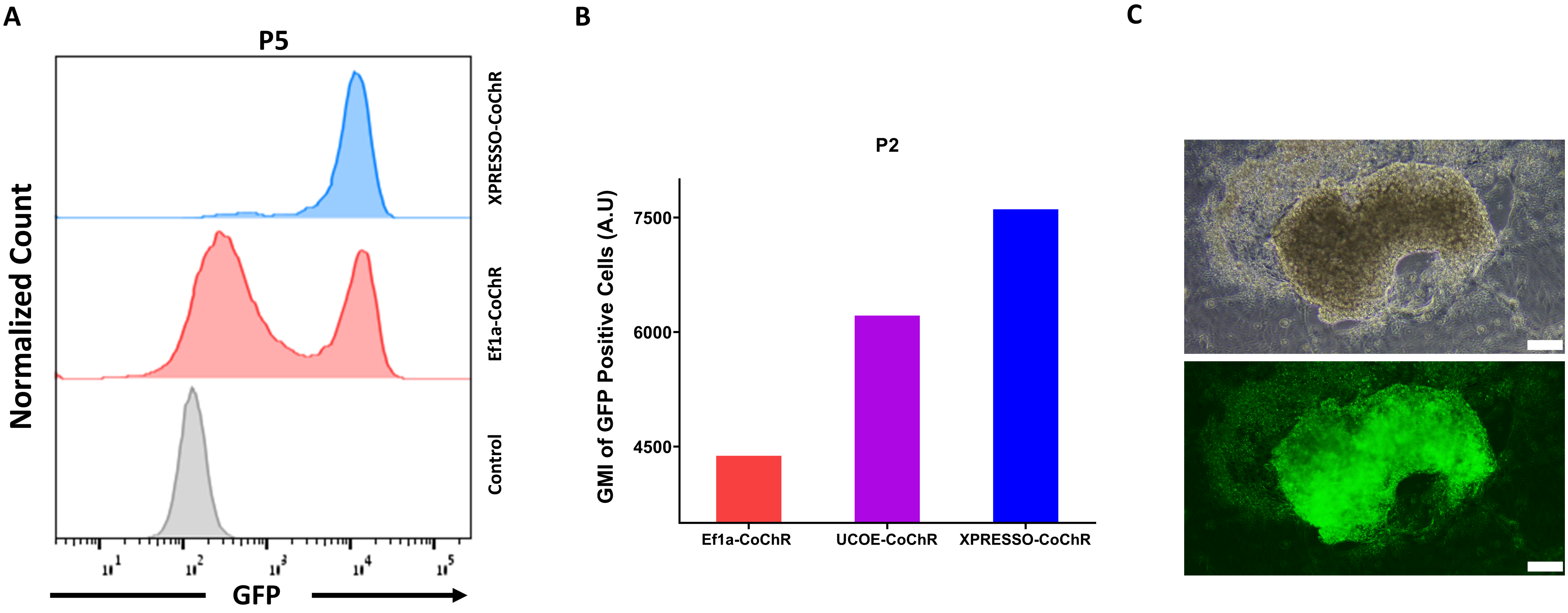

Figure S5. A. Representative offset histograms of SB-Ef1a-CoChR (red), SB-UCW-CoChR (blue), and negative control (gray) hiPSCs at P5 showing greatly improved eGFP expression in XPRESSO-CoChR compared to SB-Ef1a-CoChR hiPSCs. **B.** Bar graph comparing the geometric mean fluorescent intensity of the GFP-positive population in each of the lines at P2. A clear trend in which the intensity of GFP expression increases with each successive iteration of the vector is observable. **C.** Representative fluorescent and phase-contrast microscopy images of hiPSC-CMs differentiated from an XPRESSO-CoChR line 185 days after transfection. Strong eGFP expression was observed in the beating CMs. 10X objective, scale-bar: 100 µm

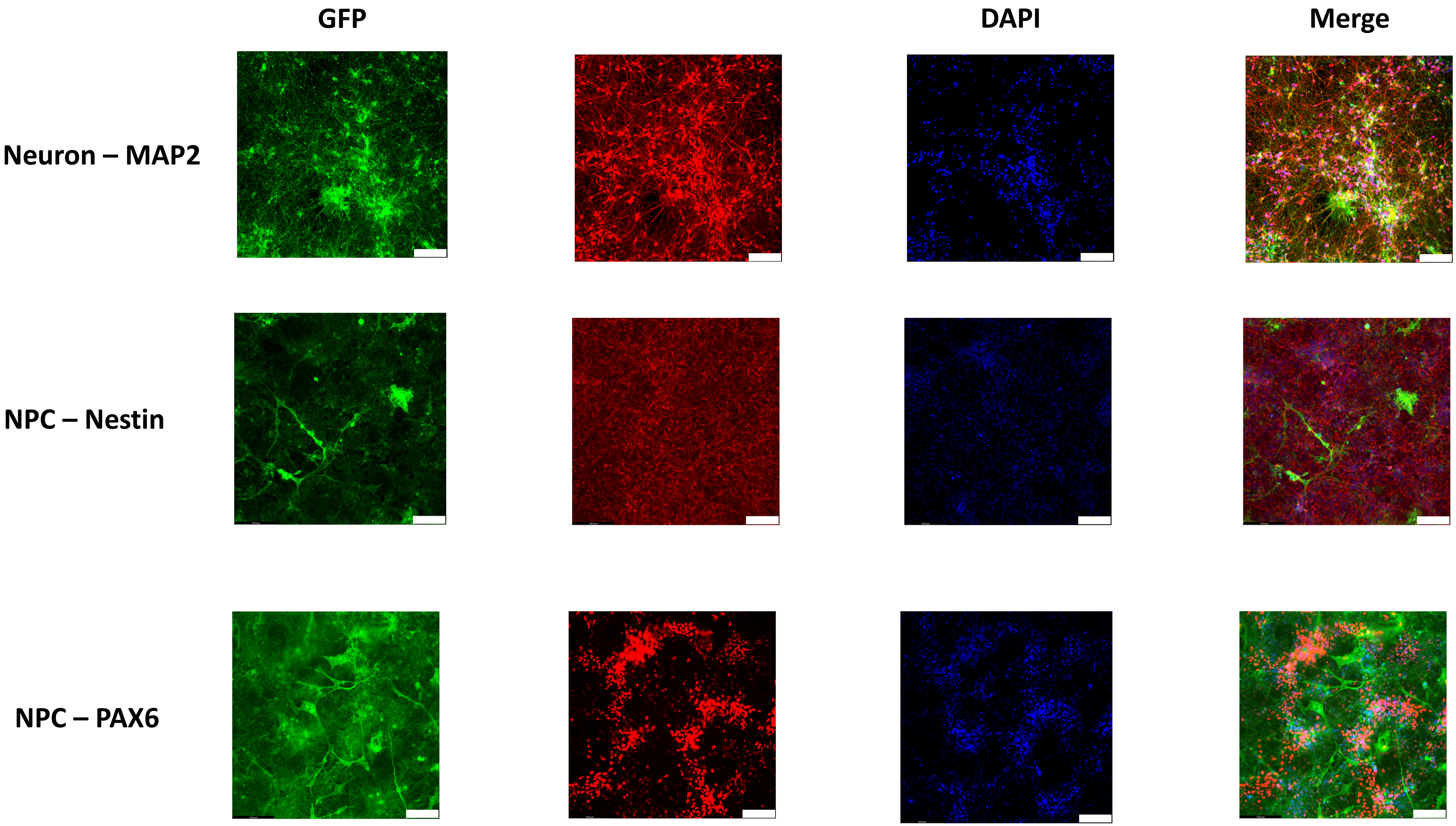

Figure S6. Immunostains of hiPSC derived neural progenitor cells (NPCs) and cortical neurons. Unique antibody used in each row is stained red. Scale-bar: 100 µm.

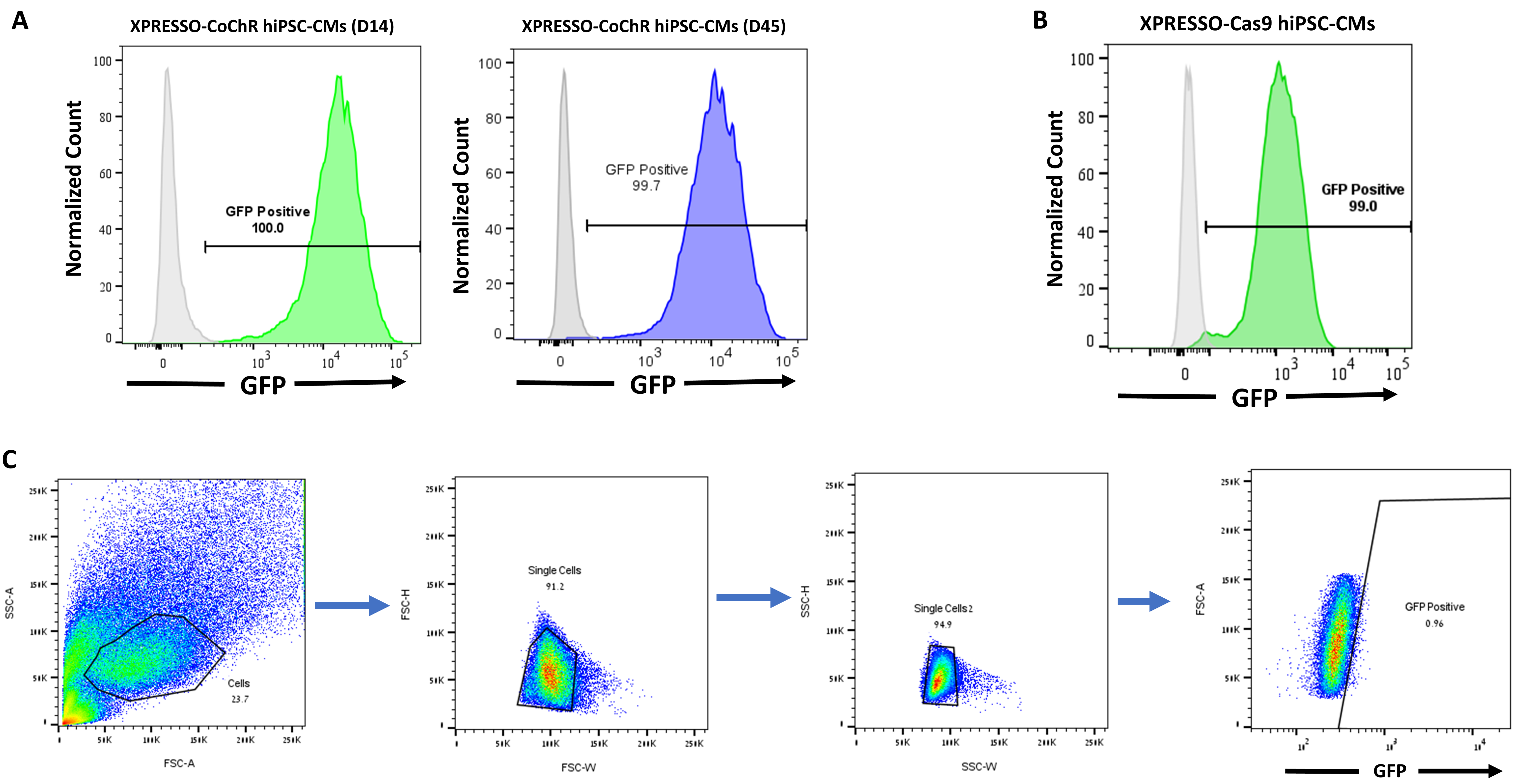

Figure S7. **A.** Representative histograms of hiPSC-CMs derived from the XPRESSO-CoChR line comparing expression 2 weeks (left) and 7.5 weeks (right) after beginning differentiation. **B.** Representative histogram of hiPSC-CMs derived from the XPRESSO-Cas9 line (passage 6). **C.** Representative flow cytometry gating strategy used to quantify the percentage of GFP positive cells in a population of control hiPSCs.

Supplemental Table S1

Method	XPRESSO (Wexler et al.)	AAV Transduction	Lentiviral Transduction	CRISPR-Cas9	TALENS and Zinc-Finger Nucleases	PiggyBac	γ-Retroviral Transduction
**Suited for overexpression**	Highly	Minimally (single copy)	Highly	Minimally (single copy)	Minimally (single copy)	Highly	Highly
**Packaging capacity**	150Kb has been demonstrated. No theoretical limit	~4.7Kb (including homology arms if needed)	~9Kb	Most commonly 3-5Kb, including homology arms	Most commonly 3-5Kb, including homology arms	200Kb shown No theoretical limit	~7.5 – 8.5Kb
**Ease of application**	Very easy. feeder free, non-viral, chemical transfection based. rapid and robust.	Labor intensive production of viruses for each gene	Labor intensive production of viruses for each gene. Requires biosafety level 2	Medium (requires PAM sequence, gRNA and donor template design)	Labor intensive generation of nucleases (for targeted)	Very easy	Labor intensive production of viruses for each gene. Requires biosafety level 2
**Time to stable line**	~1 Week	Weeks-months	Weeks-months	Months	Months	Weeks	Weeks-months
**Allows for excision**	Yes (with footprint)[2]	No	No	No	No	Yes	No
**Targetable**	Progress in targeting is being made[3]	Using homology arms or to AAVS1	No	Yes	Yes	Progress is being made	No
**Efficiency**	High	Low	High	Low or very low	Low or very low	High	High
**Viability**	High	Medium	Very low	High	High	High	Very low
**Multiplexable**	multiple genes can be inserted in each transfection by utilizing different markers or antibiotic resistance genes	Multiple viruses can be used	Multiple viruses can be used	Limited due to efficiency	Limited due to efficiency	Yes	Multiple viruses can be used
**Risk of off-target and positional effects**	Near Random insertion at TA sites, minimal preference for open reading frames[4]	Low	Highly prone to insertional mutagenesis	Off-target mutations not uncommon, gRNA dependent	Varies depending on the specific nuclease and target	Marked preference for 5' upstream of active genes and TSSs[4]	Highly prone to insertional mutagenesis
**Lab Biosafety Profile**	Excellent	Medium	Very low	Excellent	Excellent	Excellent	Very low

1) Kumar *et al.* Human Gene Therapy, 2001
2) Kesselring *et al*. Nucleic Acids Research, 2020, demonstrated how a single amino acid mutation in the SB transposase generates a unidirectional excisionase which can be used to efficiently remove transposed genes.
3) Kovac *et al.* eLife 2020, demonstrate improved targeting by fusing the SB transposase to the catalytically inactive dCas9, and providing a single guide RNA.
4) Gogol-Doring *et al.* Molecular Therapy 2016, demonstrated that the sleeping beauty transposon has the greatest safety profile in terms of random genomic insertion. This is contrasted to the PB transposon, which shows a significant preference for active genes and transcriptional start sites.

Supplemental Table S2

Primers for Amplifying GOIs

Name	Seq 5'->3'	Notes
CoChR-SfiI Fwd	gtatctGGCCtctgaGGCCgccaccgccaccatgctgggaaa	
CoChR-SfiI Rev	cgcattaGGCCtgacaGGCCGTC TCATTACTTGTACAGCTCGTCC	
EGFP-SfiI Fwd	atctGGCCtctgaGGCCgccaccatggtgagcaagggcgaggag	
EGFP-SfiI Rev	cgcattaGGCCtgacaGGCCGTCTCATTACTTGTACAGCTCGTCC	Same as CoChR-SfiI Rev
U6-shGFP-SfiI Fwd	gtatctGGCCtctgaGGCCCACCGAGGGCCTATTTCC	Used to amplify the U6 promotor and shGFP from the pLKO.1 Vector
U6-shGFP-SfiI Rev	cgcattaGGCCtgacaGGCCgtctcaCCAAAGTGGATCTCTGCTGTCC	
Cas9-EGFP-SfiI Fwd	GTATCTGGCCTCTGAGGCCGCCACCATGGCCCCAAAGAAGAAGC	
Cas9-EGFP-SfiI Rev	cgcattaGGCCtgacaGGCCgtctcacttgtacagctcgtccatgc	
Quasar2-SfiI Fwd	gtatctGGCCtctgaGGCCgccaccatgggcaagcccatccccaac	
Quasar2-SfiI Rev	cgcattaGGCCtgacaGGCCgtcttacacctcgttctcgtagc	

gRNA Sequences

Name	Target Sequence (5'->3')	Notes
sgEGFP-1	GAGCTGGACGGCGACGTAAA	Targets EGFP Nucleotide 48->
sgEGFP-2	GGTGAACCGCATCGAGCTGA	Targets EGFP Nucleotide 356->
sgKCNH2 - 1	ACCATCATCCGCAAGTTTGA	Targets first exon of KCNH2 gene
sgKCNH2 - 2	GTGTGTACGACCCAAACCCT	Targets first intron of KCNH2 gene

shRNA Sequences

Name	Sequence (5'->3')	BP	Note
shGFP_Fwd	CCGGTACAACAGCCAC AACGTCTATCTCGAGATA GACGTTGTGGCTGTTGTA TTTTTG	58	Fwd and Rev primers were annealed to create the final shGFP construct which was then cloned into the commercial pLKO.1 vector.
shGFP_Rev	AATTCAAAAATACAACAGCC ACAACGTCTATCTCGAGATA GACGTTGTGGCTGTTGTA	58	
Final shGFP Seq	AAAAATACAACAGCCACAA CGTCTATCTCGAGATAGAC GTTGTGGCTGTTGTA	53	After insertion into pLKO.1

qPCR and dPCR Primer Sequences

Name	Target Sequence (5'->3')
MYH7-Fwd	AGACTGTCGTGGGCTTGTATCAG
MYH7-Rev	GCCTTTGCCCTTCTCAATAGG
GFP-Fwd	GAGCGCACCATCTTCTTCAAG
GFP-Rev	TGTCGCCCTCGAACTTCAC
RPPH1-Fwd	AGCTGAGTGCGTCCTGTCACT
RPPH1-Rev	TCTGGCCCTAGTCTCAGACCTT
GAPDH-Fwd	ACCCACTCCTCCACCTTTGAC
GAPDH-Rev	ACCCTGTTGCTGTAGCCAAATT

Random Spacer Control Sequence:

5’->
CCGGGCAAACGCGGTCAGGAGAGAGAGCTCTAATGTTGATTGAATCGGAAATACCCCTTATCAATCAGCATTACTATTTTGAT
GTAGATAACGATGTTGAACTCATGGAGCCTATGAGTTCGTAGCTAACTGATCTTCCTGTCCACGGAGGCCCCGTCCATAAGCAA
TGATGTTGCACTCCTATGGTATGCAACTCGAGTAGATACCGACAGTGTGTCTGTTGTTGTAGCGTTCGCCTCGAGTGTACTACC
GCCAGCTGTGAGTCGGCGGGGCAGATCGTCCACTAGAAATTTAATTGGACGGTCGACTCGAATTATGGGAGAATAGCCACTC
AGCATGGACGACATATCAGATACCTCGCAAGCCCCTCTACGTATATAGCAACTGAGTCCAGGTATCAAAGTACTTACTGTTAAG
AATGACAGGCAA

Supplementary Note 1

PCR Amplification of Insert and SfiI Cloning

Primer design:

The below primer adapters create an amplified gene with a stop codon at the 3' end of the inserted gene. to change that, modify the "**tca**" sequence at the end of the reverse primer. They also create a Kozak consensus sequence at the 5' end ("gccacc"), This sequence can be omitted if it already exists at the 5' end of the gene being inserted.

A "ATG" start codon is included for amplicons that do not already include one.

Create forward and reverse primers of ~18 bp that match the 5' and 3' ends of your gene of interest (GOI), and add the adapter sequences below at the 5' end of the primer.

For Forward Primer:

gtatctGGCCtctgaGGCCgccaccATG->5' forward primer of amplicon

For Reverse Primer:

cgcattaGGCCtgacaGGCCgtc**tca**->5' of reverse primer of amplicon

PCR:

Amplify your GOI with the above primers using standard PCR (proofreading polymerase recommended). Run the resulting product on an agarose gel to insure a single band. If multiple bands are present, we recommend performing a gel-cleanup to isolate the desired band or modifying the annealing temperature to increase specificity.

Restriction:

To create a functional SB plasmid with your GOI, both the SB backbone and the insert (created with the above primers) need to be restricted in separate reactions. The following protocol is appropriate for use with the NEB SfiI restriction enzyme and the rCutSmart buffer:

X ul DNA (1 ug of Backbone/Insert)

5 ul Cutsmart buffer

44-X µl UPW

1 ul SfiI enzyme

----

50 µl Total Volume

Incubate at 50° for 1-3 hour and clean the restriction products.

Ligation:

A molar ratio of 5:1 should be used between insert:backbone. We used NEB T4 ligase in a 20 ul reaction volume incubated overnight at 16°, and heat inactivated at 65° for 15 minutes.

Transformation:

The ligation product can be directly transformed into competent bacteria, and resulting colonies screened with PCR.

DNA Preparation:

Positive colonies should be inoculated in LB and prepped using standard kits. Mini-preps are sufficient for multiple transfections as only ~1.5-1.7 ug of plasmid DNA are necessary per transfection.

Electroporation of SB plasmids into hiPSCs

We performed electroporation in to hiPSCs using the NEPA21 Super Electroporator (NEPAGENE).

After careful calibration of the electroporation parameters, the following protocol led to the greatest transfection efficiency and survival of electroporated hiPSCs.

1. Disassociate and count hiPSCs and create 1.5 million cell aliquots in 100ul of OptiMEM.
2. Immediately before electroporation add 15-20ug of plasmid DNA (3:1 transposon:transposase ratio) to each aliquot (added volume should be less than 15ul).
3. Transfer each aliquot to the electroporation cuvette, insuring there are no bubbles.
4. Insert the cuvette into the electroporator and assess the resistance, the range should be between 0.033-0.053. If the resistance is too high, add Opti-MEM until the correct range is achieved.
5. Program the electroporator according to the parameters below and execute the electroporation.
6. Immediately after electroporation, add 100ul of hiPSC growth medium with supplemented ROCK inhibitor to the cuvette.
7. Transfer the contents of the cuvette into a 35mm, Matrigel coated, well containing 2ml of growth medium supplemented with ROCK inhibitor.
8. Assess fluorescence after 24 hours and begin selection with appropriate antibiotic.

Set Parameters											
Poring Pulse						Transfer Pulse					
V	Length (ms)	Interval (ms)	No.	D. Rate (%)	Polarity	V	Length (ms)	Interval (ms)	No.	D. Rate (%)	Polarity
**125**	**5**	50	2	10	+	20	50	50	5	40	+/-

Supplemental Methods

Propagation and cardiomyocyte differentiation of hPSCs

Modified hESC (Protze et al., 2017) and healthy-control hiPSC (Itzhaki et al., 2011) lines that were previously established and described were used. Undifferentiated stem cell colonies were grown on feeder-free Cultrex (R&D Systems cat # 3433-010-01) coated plates using mTeSR1 medium. Nearly confluent wells were passaged twice weekly following incubation with EDTA (0.5 mM) and replated with Thiazovivin (Cayman Chemicals cat #14245) at a final concentration of 2 µM. Cells were frozen using NutriFreez™ D10 Cryopreservation Medium (Sartorius cat # 05-713-1B) and thawed according to the manufacturer's protocol. Sterility was assessed daily, and mycoplasma was tested for quarterly. For differentiation of PSCs to cardiomyocytes, lines from passage 2-10 were used unless otherwise stated, except for the Ef1α-CoChR line in which passages 2-5 were used. A modified monolayer directed differentiation protocol was used as previously described (Shinnawi et al., 2015). Briefly, RPMI 1640 with 2% b27 minus-insulin supplement and 1% penicillin/streptomycin was used for differentiation. From day 0-1 6 mM CHIR99021 (Stemgent) was added. On days 2-3, 2 µM Wnt-C59 (Selleckchem) was added, without CHIR99021. Beating cardiomyocytes were observed on days 9-14 of differentiation, enzymatically disassociated using TrypLE (Thermo Fisher Scientific) between days 12-25 and replated as CCSs or single cells on Cultrex-coated plates.

Cortical neuronal progenitor cells (NPCs) differentiation

CoCHR-GFP-WPRE hiPSCs were cultured in mTeSR plus (Stem cell technologies) in Matrigel coated 6 well plates (Corning, cat# 07-200-83). Cortical NPCs were generated as described (Hussein et al., 2023). Briefly, hiPSCs were grown until they reached 75-80% confluency. They were washed with DPBS and treated with dispase 1 U/ml (Stem cell technologies, Cat # 07923) for 15-20 minutes. The dispase was then aspirated and the hiPSCs were scraped gently, collected, and allowed to settle in a 50 ml conical tube. After 15 minutes, the supernatant was removed and gently resuspended in mTeSR plus media with ROCK inhibitor Y-27632 (Enco cat # 129830-38-2). The cells were then transferred to a low attachment dish (Corning ultra-low attachment cat# CLS3261) and maintained in Embryoid Bodies (EBs) media containing DMEM-F12 (Thermo Fisher scientific cat# 11320033), Glutamax 1X (Thermo Fisher scientific, cat# 35050061), B27 1X (Thermo Fisher scientific, cat# 17504044), N2 1X (Thermo Fisher scientific, cat# 17502048), and LDN193189 hydrochloride 0.1 μM (Peprotech cat# 1066208). After 10 days, the cortical EBs were transferred onto Poly-l-ornithine (Merck, cat# P4957)/Laminin (Thermo Fisher scientific, cat# 23017015) coated 6 well plates and allowed to attach and form neuronal rosettes in EB media with an addition of laminin 1 μg/ml (Thermo Fisher scientific, cat# 23017015). The neuronal rosettes were then selected, picked, and treated with StemPro™ Accutase™ (Thermo Fisher scientific, cat# A1110501) for dissociation into single cells. After 10 min, the DMEM-F12 with trypsin inhibitor (Sartorius, cat # 03-048-1C) was added and the cells were further dissociated with gentle pipetting. The cells were plated in NPC medium containing DMEM-F12, Glutamax 1X, human FGF-2 20ng/ml (Peprotech cat # AF-100-18B), B27 1X, N2 1X, and laminin 1 μg/ml.

Cortical neuron differentiation from NPCs

For differentiation into cortical neurons, cortical NPCs in 6 well plates were treated with Accutase™ for 5 minutes. After 5 minutes, the NPCs were dissociated, washed, and collected in wash media composed of DMEM-F12 and 1X Glutamax. The dissociated cortical NPCs were counted using a Trypan blue exclusion assay with Bio-Rad TC20™ automated cell counter. Approximately 1 million cells were seeded on Poly-l-ornithine/laminin-coated 6-well tissue culture plates. After 24-36 hours, the cortical NPCs were differentiated using the primary neuronal differentiation media containing DMEM-F12 with 1X Glutamax, 1X B27 supplement, 1X N2 supplement, 0.2 nM Ascorbic Acid (Stem cell technologies, cat# 72132), 500 μg/ml cyclic-AMP (TOCRIS, cat# 1141), 20 ng/ml BDNF (Peprotech, cat# 450-02), 20 ng/ml GDNF (Peprotech, cat# 450-10), and 1 μg/ml laminin for 10-12 days. Next, the cortical neurons were dissociated again with Accutase™ and Trypsin inhibitor as described above for cortical NPC dissociation. The cortical neurons were gently dissociated with mechanical pipetting and approximately 700K cortical neurons were seeded onto Poly-l-ornithine/Laminin coated 13 mm glass coverslips (Fisher Scientific, cat# 10513234) in 24 well tissue culture plates (Corning, cat# 09-761-146) in the Primary neuronal differentiation media with Rock inhibitor. After 24-36 hours, half of the primary neuronal differentiation media was replaced by secondary neuronal differentiation media composed of BrainphysTM (Stem cell technologies cat# 05790), 1X B27 supplement, 1X N2 supplement, 0.2 nM Ascorbic, 500 μg/ml cyclic-AMP, 20 ng/ml BDNF, 20 ng/ml GDNF, and 1 μg/ml laminin. The cortical neurons were thereafter maintained in

secondary neuronal differentiation media with half media changes every other day for ~2 months until electrophysiology and immunocytochemistry experiments were performed.

Plasmid construction, propagation, and harvesting

The original SB transposon plasmid pSBbi-RP was a gift from Eric Kowarz (Kowarz et al., 2015) (Addgene plasmid #60513) and the SB100X transposase plasmid pCMV(CAT)T7-SB100 was a gift from Zsuzsanna Izsvak (Mátés et al., 2009) (Addgene plasmid #34879). To generate the UCOE-SB plasmid backbone, we synthesized a minimal UCOE element (Synbio Technologies) previously described (Zhang et al., 2017), and used restriction enzyme cloning to insert it upstream of the EF1α promoter. To generate the XPRESSO vector, we used the modified UCOE-SB vector backbone and restriction cloning to remove the EF1α promoter and insert the CAG promoter, which was excised from the pCAGIG plasmid, a kind gift from Connie Cepko (Matsuda and Cepko, 2004) (Addgene plasmid #11159). Finally, we inserted the WPRE element downstream of the stop codon using restriction cloning. We inserted all the transgenes of interest into the different SB vector types using dual SfiI restriction enzyme sites. In shRNA SB vectors, the U6 promoter was added upstream of the shRNA sequences (see **Supplementary table S2**). We used PCR amplification to amplify the inserts and add SfiI sites to the ends. The CoChR transgene was kindly provided by Ofer Yizhar (Weizmann Institute). The CaViar gene was amplified from the pJMK074: CMV QuasAr2-TS-GCaMP6f plasmid, which was a gift from Adam Cohen (Dempsey et al., 2016) (Addgene plasmid #72303). The Cas9-T2A-eGFP gene was amplified from pSpCas9(BB)-2A-GFP (PX458), which was a gift from Feng Zhang (Ran et al., 2013) (Addgene plasmid #48138). A simplified

protocol for the design of primers with adapter sequences followed by cloning instructions can be found in **Supplementary note 1**.

STBL4 (Thermo Fisher scientific) or DH5α bacteria were used to propagate the plasmids. Plasmid DNA was extracted via miniprep (NucleoSpin Plasmid, Mini Kit) from 4ml of overnight LB cultures or via midiprep (PureLink™ HiPure Plasmid Midiprep Kit) from 100-200ml of overnight LB cultures if greater amounts of DNA were necessary.

Transfection and antibiotic selection

Nearly confluent hPSCs were passaged the day before transfection into 35mm diameter wells (6-well plate) so that the cells would be 15-40% confluent after 24 hours. Using a modified version of a previously reported protocol (Giacalone et al*.*, 2018), transfection wells were aspirated 30 minutes before transfection and 1.5 ml of mTeSR1 medium was added. The transposon and transposase plasmids were mixed in a 3:1 molar ratio of transposon:transposase, so that a total of 2 μg of plasmid DNA was used. The DNA was added to 50ul of Opti-MEM (Gibco) and vortexed. Next, 12.5 μl of Lipofectamine Stem (Thermo Fisher scientific) was added to the DNA and the mixture was incubated at room temperature for 10-15 minutes. Following incubation, the mixture was added dropwise to each well. Electroporation of SB plasmids was also found to be highly efficient and may be used instead (see **Supplementary note 1** for a detailed electroporation protocol). Puromycin (0.8-1 μg/ml) was added to the cells 24 hours after transfection, upon observation of fluorescence. Selection medium was replaced daily, besides for on the weekends. Once positive colonies were easily distinguishable (day 4-7 after transfection),

either the entire well was disassociated and replated to create a heterogeneous genetically modified population, or single colonies were picked to create clonal lines. After colony picking, or replating of a heterogeneous population, cells were grown in selection medium for an additional 24 to 48 hours and grown in mTeSR1 medium without puromycin from then on.

Flow cytometry and analysis

Flow cytometry was performed on live and fixed cells using a BD LSR Fortessa II cytometer. Cells were disassociated and pelleted. For live cell cytometry, cells were washed once with PBS, pelleted, resuspended in PBS, and filtered through a strainer cap. For fixed cell cytometry, cells were washed once with PBS, incubated with PFA 2% for 10 minutes at 4 degrees, washed twice more with PBS, resuspended in PBS, and filtered through a strainer cap. Analysis was performed using FlowJo™ Software (BD Life Sciences). The gating strategy can be seen in **Supplementary figure S7**.

Immunostaining and microscopy

Undifferentiated hiPSC and differentiated hiPSC-CMs, 15-30 days after differentiation, were fixed in 4% PFA (Bio-Lab) for 20 minutes, permeabilized with 1% Triton X-100 for 10 min (Sigma-Aldrich) and blocked with 5% horse serum (Gibco) for one hour, all at room temperature. Cells were incubated with primary antibodies overnight at 4°, washed three times, and incubated for 1 hour in the dark at room temperature with secondary antibodies. Cell nuclei were counterstained using DAPI (1:500, Sigma-Aldrich, D9564). Cells were imaged using a confocal microscope (Zeiss LSM900) or a fluorescent microscope (Zeiss

Celldiscover 7 or Zeiss Primovert iLED) and the appropriate fluorescent filter sets. Primary antibodies used targeted α-Actinin (1:150, Sigma cat# A7811), cardiac troponin T (1:150, abcam cat# 91605). Secondary antibodies were Cy3 donkey anti-mouse IgG (1:200, Jackson ImmunoResearch cat# 715-165-151), and Cy5 donkey anti-rabbit IgG (1:200, Jackson ImmunoResearch cat# 711-175-152). For live cell staining, NucBlue (Thermo Fisher scientific) according to the manufacturer's protocol.

Immunostaining of differentiated cortical NPCs was performed at 3-4 days when confluent and cortical neurons at 30 days of differentiation. Cells were fixed in 4% paraformaldehyde for 15 minutes at 37°C followed by three DPBS washes. Cortical NPCs were immunostained with Nestin (Cell Signaling Technologies, cat #33475) and Pax6 (Cell Signaling Technologies, cat #60433) and cortical neurons were stained with MAP2 (Abcam, cat# ab92434). The z-stack images of Neurons were acquired using a Nikon A1R Confocal Laser Scanning Inverted Microscope at 20X (1.5X magnification) and 60X (oil immersion objective). For NPCs, z-stack images were acquired on DMi8 Leica thunder imager at 20X. The confocal images for neurons were processed using Imaris 9 viewer. The NPCs images were processed using ImageJ.

Patch Clamp Analysis

Traces were recorded using the MultiClamp 700B and Digidata 1440A (Axon Instruments). Data were sampled at 20 kHz and analyzed using Clampfit 10.7 (Molecular Devices).

Cardiomyocytes: Whole-cell patch-clamp recordings were performed at room temperature in a modified Tyrode's solution composed of (in mmol/L): 3 KCL, 140 NaCl, 10

HEPES, 10 glucose, 2 CaCl2, and 2 MgCl2 (pH adjusted to 7.4 with NaOH). The recording micropipettes (tip resistance of 2-4 MΩ) were filled with an internal solution containing (in mmol/L): 120 KCL, 1 MgCl2, 3 MgATP, 10 HEPES, 10 EGTA (pH adjusted to 7.2 with KOH). For optogenetic current measurements, hiPSC-CMs were held at -60mV and stimulated with 470nm light from an LED (CoolLed pE-4000). For optogenetic action-potential measurements, the current-clamp mode was used. If necessary, cells were injected with a constant current to maintain a resting membrane potential of ~-60mv. The cells were then stimulated with 470nm LED light for varying lengths of time to elicit action potentials and observe the effect of continual illumination on its properties.

Neurons: Whole-cell patch-clamp recordings were performed on cortical neurons derived from the XPRESSO-CoChR line. Neuronal cultures on coverslips were placed in a recording chamber on an upright microscope fitted with a ×40 water-immersion objective (Slicescope pro 2000, Scientifica). The bath contained artificial cerebrospinal fluid (ACSF) containing (in mM) 139 NaCl, 10 HEPES, 4 KCl, 2 CaCl2, 10 D-glucose, and 1MgCl2 (pH 7.5, osmolarity adjusted to 310 mOsm) that had been warmed to 37˚C. The recording micropipettes (tip resistance of 10-15 MΩ) were filled with an internal solution containing (in mM) 130 K-gluconate, 6 KCl, 4 NaCl, 10 Na-HEPES, 0.2 K-EGTA, 0.3 GTP, 2 Mg-ATP, 0.2 cAMP, 10 D-glucose, 0.15% biocytin, and 0.06% rhodamine (pH 7.5, osmolarity adjusted to 290-300 mOsm). Stimulation of cells was carried out by 488 nm wavelength laser beam from a FiberTEC II Fiber-coupled Diode Laser Module (BSR FiberTec) that was powered by a Chromalase power supply (Fibotec). The light intensity was controlled and manipulated by connecting the laser to a MultiClamp 700B amplifier (Molecular Devices) and injecting

currents at different levels using the MultiClamp 700B software. Recordings in current and voltage-clamp configurations were recorded using MultiClamp 700B amplifier.

Evoked optogenetic neuronal action potentials:

Patched cells were held at -60 mV in current clamp with a constant holding current. Next, the cells were stimulated by a 488 nm laser at five increasing intensities and resulting evoked potentials were analyzed. Neurons that required more than 50 pA of holding current to maintain a voltage of -60 mV were excluded from the analysis.

Evoked optogenetic neuronal currents:

Patched neurons were held at -60 mV in voltage-clamp mode. Next the cells were stimulated by a 488 nm laser for several seconds to elicit a depolarizing current. Peak current was normalized to each cell's capacitance and used for analysis.

Generation of hiPSC-derived cardiomyocyte cell sheets (CCSs), optical mapping, and optogenetic interventions

CCSs were generated as previously described (Shaheen et al., 2018). In brief, hiPSC-CMs were enzymatically dissociated between day 12-25 of differentiation and reseeded in 50 µl drops containing between 700K-1.2M cells. CCSs were cultured in RPMI-B27 minus insulin, (-)-blebbistatin (3.3 µM), and 1% penicillin/streptomycin, and were optically mapped on days 5-7 from seeding.

Optical mapping was performed using a high-speed EM-CCD camera (Evolve 512Delta, Photometrics, 512 × 512 pixels) mounted on a macroscope (MVX10, Olympus). The CCSs

were incubated at room temperature for fifteen minutes with the voltage dye Di4-ANBDQBS according to the manufacturer's protocol (Potentiometric Probes) and excited using an LED (X-Cite TURBO, Excelitas Technologies) with a peak wavelength of 630nm. A Chroma ET620/60× filter was used for excitation and ET665lp for emission. Video acquisition was performed using Micro-Manager software, and optical maps and APD calculations were generated using OMProCCD software, a custom-made IDL based software (provided by Bum-Rak Choi, Brown University, Providence, Rhode Island, USA). Phase maps were produced using a semi-automated custom-written MATLAB script that generated color-coded phase maps as previously described (Shaheen et al., 2018).

For targeted and diffuse optogenetic stimulation of the CCSs a digital micromirror device (DMD, Polygon-400, Mightex Systems) was driven by a 470nm LED (BLS-series High-Power Light Guide Coupled LED Source, Mightex Systems). Illumination patterns were created and projected using the Polyscan2 software (Mightex Systems). Rotor-like arrhythmias were induced using electrode tachypacing (10 Hz), and diffuse illumination was used in the attempts to terminate arrhythmias.

qPCR analysis

RNA was derived from the samples using the NucleoSpin RNA extraction kit (Macherey-Nagel cat# 40955), according to the manufacturer's protocol. Reverse transcription was performed using All-In-One 5X RT MasterMix (abm cat# G592) according to the manufacturer's protocol. Real-time qPCR was performed in triplicate for each biological sample using LightCycler 480 SYBR Green I Master (Roche cat# 04707516001) in the

StepOne plus Real-Time PCR machine (Applied Biosystems) using primers for GFP and MHC (see **Supplementary table S2**), under the following conditions: 5 minutes at 95°, 40 cycles of 95° for 10 seconds, 53° for 20 seconds, and 72° for 10 seconds, followed by the performance of a melt curve.

For measurement of shRNA mediated knockdown, the cardiac MHC gene was used for normalization, and quantitative comparison of GFP mRNA transcripts was compared using the $2^{-\Delta\Delta CT}$ method. The average expression within the control line was normalized to 1, and the knockdown is presented as the percentage of expression of each biological replicate compared to the average expression of the control line.

dPCR Analysis

DNA was extracted from hiPSCs using the DNeasy Blood and Tissue Kit (Qiagen cat# 69504) according to the manufacturer's protocol. Digital PCR was performed using QIAcuity EG PCR Kit (Qiagen cat# 250111) and the QIAcuity One dPCR machine (Qiagen) according to the manufacturer's protocol and with concomitant DNA restriction using EcoRI under the following conditions: 2 minutes at 95°, 40 cycles of 95° for 15 seconds, 60° for 15 seconds, and 72° for 15 seconds, followed by 5 minutes at 40°. The reaction was performed using primers for a single-copy gene (RPPH1) and for eGFP (Kolacsek et al., 2011) (see **Supplementary table S2**) and copy number quantification was calculated by dividing the absolute concentration of eGFP by the concentration of RPPH1 and multiplying by 2 (to account for the two copies of RPPH1 per human diploid genome).

Calcium Imaging

Live whole-cell calcium imaging was performed on differentiating colonies using the above-mentioned optical mapping setup (Olympus MVX10) and excitation with an LED with peak wavelength of 475 nm (X-Cite TURBO, Excelitas Technologies). The recorded signals were filtered and analyzed using Micro-Manager software. For disassociated single cells, calcium imaging was performed using a Zeiss LSM900 confocal microscope. Individual spontaneously beating cardiomyocytes derived from the SB-CaViar hiPSC line were measured for fluorescent intensity over time using the line scan function and the GFP filter set. The resulting signal was analyzed and smoothed using GraphPad Prism 9.

gRNA Transfection

In a 12-well plate, XPRESSO-Cas9 hiPSCs were transfected at ~20-40% confluence with two sgRNAs targeting eGFP/KCNH2 (sgGFP1: GAGCTGGACGGCGACGTAAA, sgGFP2: GAGCTGGACGGCGACGTAAA, 1 μg of each) or with 2 μg of negative control scramble gRNA using Lipofectamine CRISPRMAX (Thermo Fisher scientific) according to the manufacturer's instructions. The wells were passaged 2 days after transfection and eGFP expression was assessed using flow cytometry six days after transfection. For the KCNH2 KO experiment, XPRESSO-Cas9 hiPSC-CMs were disassociated and replated in 12-well plates at 85-100% confluence and transfected in the same manner with 2 μg of KCNH2-targgeting sgRNAs (sgKCNH2-1: ACCATCATCCGCAAGTTTGA, sgKCNH2-2: GTGTGTACGACCCAAACCCT) or scramble gRNA. All sgRNAs were synthesized by IDT technologies.

Statistical analysis

All statistical analyses were performed using the Prism GraphPad 9 software. All values are displayed as mean ± SEM. When assessing differences between two groups of continuous variables, two-sided paired or unpaired Student's *t* tests were used. When the groups were composed of discrete variables, Fisher's exact test was used. When comparing multiple groups with a single independent variable, one-way ANOVA with multiple comparisons using Tukey's correction was applied. When tracking the different engineered lines over multiple passages, a two-way ANOVA with Šidák's correction for multiple comparisons was used. When any data points for comparison were missing, a Mixed-effects analysis using the Geisser-Greenhouse correction and Tukey's correction for multiple comparisons was used instead. All p-values<0.05 were considered statistically significant.
